# A DNA-Methylation-Driven Genes Based Prognostic Signature Reveals Immune Microenvironment in Pancreatic Cancer

**DOI:** 10.3389/fimmu.2022.803962

**Published:** 2022-02-10

**Authors:** Mingjia Xiao, Xiangjing Liang, Zhengming Yan, Jingyang Chen, Yaru Zhu, Yuan Xie, Yang Li, Xinming Li, Qingxiang Gao, Feiling Feng, Gongbo Fu, Yi Gao

**Affiliations:** ^1^Department of Hepatobiliary Surgery II, Zhujiang Hospital, Southern Medical University, Guangzhou, China; ^2^Ultrasound Medical Center, Zhujiang Hospital, Southern Medical University, Guangzhou, China; ^3^Department of Pathology, School of Basic Medical Sciences, Southern Medical University, Guangzhou, China; ^4^First College of Clinical Medicine, Southern Medical University, Guangzhou, China; ^5^Department of Critical Care Medicine, Zhujiang Hospital, Southern Medical University, Guangzhou, China; ^6^Department of Radiology, Zhujiang Hospital, Southern Medical University, Guangzhou, China; ^7^Department of Biliary Surgery I, Eastern Hepatobiliary Surgery Hospital, Shanghai, China; ^8^Department of Medical Oncology, Affiliated Jinling Hospital, Medical School of Nanjing University, Nanjing, China; ^9^State Key Laboratory of Organ Failure Research, Southern Medical University, Guangzhou, China

**Keywords:** pancreatic cancer, DNA methylation driven gene, prognostic signature, tumor immune, precision medicine

## Abstract

Pancreatic cancer (PACA), which is characterized by an immunosuppressive nature, remains one of the deadliest malignancies worldwide. Aberrant DNA methylation (DNAm) reportedly influences tumor immune microenvironment. Here, we evaluated the role of DNA methylation driven genes (MDGs) in PACA through integrative analyses of epigenomic, transcriptomic, genomic and clinicopathological data obtained from TCGA, ICGC, ArrayExpress and GEO databases. Thereafter, we established a four-MDG signature, comprising GPRC5A, SOWAHC, S100A14, and ARNTL2. High signature risk-scores were associated with poor histologic grades and late TNM stages. Survival analyses showed the signature had a significant predictive effect on OS. WGCNA revealed that the signature may be associated with immune system, while high risk-scores might reflect immune dysregulation. Furthermore, GSEA and GSVA revealed significant enrichment of p53 pathway and mismatch repair pathways in high risk-score subgroups. Immune infiltration analysis showed that CD8+ T cells were more abundant in low score subgroups, while M0 macrophages exhibited an opposite trend. Moreover, negative regulatory genes of cancer-immunity cycle (CIC) illustrated that immunosuppressors TGFB1, VEGFA, and CD274 (PDL1) were all positively correlated with risk-scores. Furthermore, the four signature genes were negatively correlated with CD8+ lymphocytes, but positively associated with myeloid derived suppressor cells (MDSC). Conversely, specimens with high risk-scores exhibited heavier tumor mutation burdens (TMB) and might show better responses to some chemotherapy and targeted drugs, which would benefit stratification of PACA patients. On the other hand, we investigated the corresponding proteins of the four MDGs using paraffin-embedded PACA samples collected from patients who underwent radical surgery in our center and found that all these four proteins were elevated in cancerous tissues and might serve as prognostic markers for PACA patients, high expression levels indicated poor prognosis. In conclusion, we successfully established a four-MDG-based prognostic signature for PACA patients. We envisage that this signature will help in evaluation of intratumoral immune texture and enable identification of novel stratification biomarkers for precision therapies.

## Introduction

Pancreatic cancer (PACA) is a highly fatal malignancy, with a 5-year survival rate of less than 10% (1). The current classical TNM staging system as well as biomarkers, such as CA 19-9 and CA 125, are not efficient and accurate enough in diagnosing and predicting prognosis of patients with PACA. Recent genomic profiling studies have revealed tremendous heterogeneity in PACA and potentially actionable gene alterations in small subsets of patients, implying the feasibility of targeted therapies or immunotherapies (2, 3). However, results from earlier clinical trials on immunotherapies for blocking PD1/PD-L1 in PACA patients did not yield encouraging results, with the obtained poor responses attributed to immunosuppressive conditions in the PACA tumor microenvironment (TME), including a scarcity of CD8+ T cells and a recruitment of myeloid cells, respectively (4).

Epigenetic changes have long been reported to play important roles in carcinogenesis, tumor progression as well as immune escapes. DNA methylation (DNAm), a major type of epigenetic alterations (5), has been shown to alter promoter regions by methylating CpG dinucleotides, thereby causing gene silencing, including some tumor suppressor genes (6). RNA modifications, especially N6-methyladenosine (m6A), confer malignant cells with the abilities to reversibly alter their transcriptional profiles rapidly and reversibly in order to survive the stressful microenvironment (7). Deregulated DNAm can act as an early diagnostic and prognostic biomarker, suggesting its potential for the management of various cancers (8, 9). Besides, tumor immunogenicity and immune cells, as long as anti-tumor responses, are reportedly influenced by DNAm and m6A (10, 11). Epigenetic changes in PACA have been reported to influence the immune microenvironment and patient outcomes (12, 13). However, due to the complexity and obscurity of epigenetics, it is difficult to interpret the biological effects of these epigenetic biomarkers. We hypothesized that due to the regulatory relationship between DNAm and gene expressions, identification of methylation-regulated differentially expressed genes, also referred to as methylation-driven genes (MDGs), and assessment of their features may help in elucidating the characteristics of PACA.

Therefore, we performed a comparative integrated analysis of transcriptome, DNAm and clinical data from TCGA and ICGC datasets to identify prognostic MDGs in PACA. Then, we used these markers to establish and validate a predictive signature across all included datasets from TCGA, ICGC, GEO and ArrayExpress databases. Finally, based on this signature, we evaluated the molecular characteristics of PACA subgroups, especially its correlation with immune TME, as well as its utility in therapeutic response prediction.

## Materials and Methods

**Figure 1:**
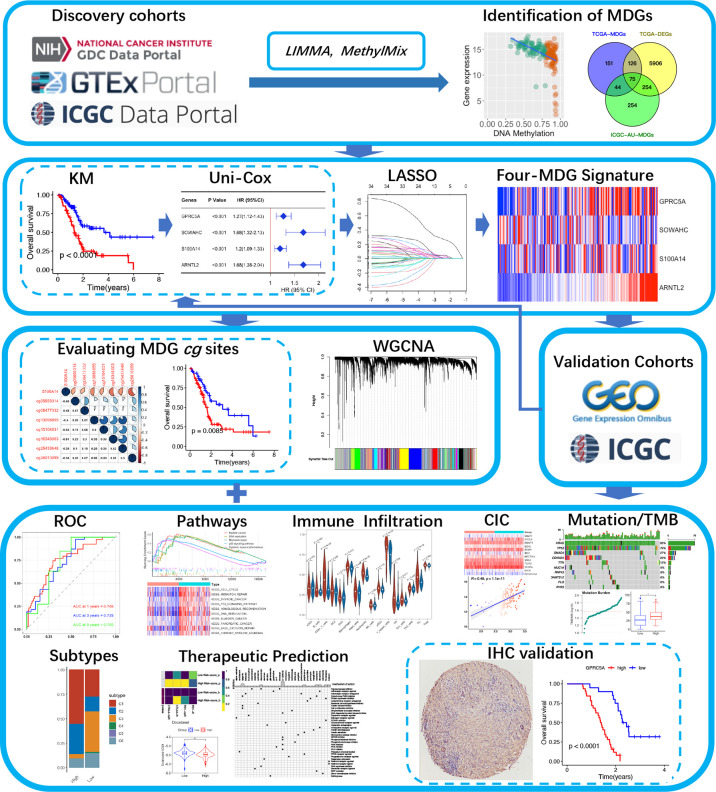
A flow chart of the current study.

A schematic presentation of the research procedure is shown in **Figure 1**.

### Data Acquisition and Preprocessing

Transcriptomic data, DNAm data, somatic mutation data and clinicopathological data of the TCGA-PAAD project were downloaded from the TCGA database. Differential expression analysis was performed using an integrated TOIL GTEx and TCGA transcriptic dataset, obtained from the UCSC Xena database (https://xena.ucsc.edu/). The workflow software, Toil, was used to reprocess raw data in GTEx and TCGA to correct batch effects as well as merge data for pan-analyses (14). Transcriptomic, DNAm and clinical data of ICGC-PACA-AU (15) and ICGC-PACA-CA (16) projects were obtained from the ICGC database. Summarily, the ICGC-PACA-AU project contains RNA sequencing data (seq) and microarray data (array) which were used as individual datasets according to specific analyses, whereas the ICGC-PACA-CA dataset contains a lot of “NA” values either in the expression matrix or in the clinical data, which made it unsuitable for some subsequent analyses. Besides, level 3 data of GSE62452, GSE78229, and E-MTAB-6134 datasets were downloaded from the GEO and ArrayExpress databases, using the following inclusion criteria: (1) PACA transcriptomics studies either by sequencing or microarray, (2) Studies analyzing adult participants (older than 18 years), (3) Study participants or samples were not restricted to some specific type, such as long survival or vascular invasions among others, (4) Sample sizes were over 30.

### Screening for Differentially Expressed Genes and Functional Enrichment of MDGs

Differentially expressed genes (DEGs) between tumor and normal tissues from TCGA-PAAD and GTEx datasets were screened using the “limma” package implemented in R, with thresholds of p < 0.05 and |log2FC| > 1. Methylation-driven genes (MDGs) are hypo and hypermethylated genes that are predictive of transcription and thus functionally relevant for a particular disease. Identification of MDGs was achieved by integrating DNAm and mRNA sequencing data from TCGA-PAAD and ICGC-PACA-AU projects, using the “MethylMix” R package. According to the instructions by the authors, MethylMix integrates DNA methylation from normal and tumorous samples and matched gene expression data *via* a three-step algorithm (17, 18). (1) Genes are filtered by identifying transcriptionally predictive methylation. (2) The methylation states of a gene are identified using univariate beta mixture modeling approach to identify subgroups of patients with similar DNA methylation level for a specific CpG site (19). (3) Hyper and hypomethylated genes are defined relative to normal by comparing the methylation levels of each methylation state to the mean of the DNA methylation levels of normal tissue samples using a Wilcoxon rank sum test. Differentially expressed MDGs, present at the intersection of DEGs and MDGs, as well as all DEGs were subjected to functional enrichment analyses by GO and KEGG, using the ConsensusPathDB databases.

### Construction of an MDG-Based Prognostic Signature

The TCGA-PAAD cohort was set as the training cohort. To determine the relationships between MDGs and overall survival (OS) of PACA patients, first, we subjected the differentially expressed MDGs to K-M analysis (Log-rank test) and univariate Cox regression analyses, then, statistically significant genes were selected and subjected to least absolute shrinkage and selection operator (LASSO) regression analysis to filter signature genes using the “glmnet” package to filter signature genes (20). Three-fold cross-validation and 1000 iterations were conducted to reduce the potential instability of the results. The optimal tuning parameter λ was identified *via* 1-SE (standard error) criterion. Then, a prognosis classifier was developed based on the individual-level risk scores derived from the selected prognostic MDG signature. For each patient, the risk score was a sum of the products of the expression levels of the prognostic signature MDGs and the corresponding regression coefficients (β) derived from LASSO model.

### Correlation Between Signature Gene Expression and Methylation

Correlations between methylation and mRNA expression levels of the signature genes were evaluated by the “corrplot” package, while the relationships between DNAm and OS were determined by K-M survival and univariate Cox analyses. The latest data on the Illumina Human Methylation 450K platform, including IllmnID (probe ID), UCSC RefGene Names (related gene symbols) and UCSC RefGene Group (Functional genomic distribution, FGD) were obtained from the official website (https://support.illumina.com).

### Assessment of the Predictive Power of the Established Signature

To validate our prediction model, we used K-M analysis and time-dependent receiver operator characteristic (ROC) curves (21) to evaluate their predictive effects in validation datasets. To identify whether our four-MDG signature depended on other clinicopathological factors, such as age, gender, AJCC 7^th^ TNM stage, and histologic grade, in predicting OS, we performed univariate and multivariate Cox regression analyses using the “survival” package, then analyzed the resulting correlations. Moreover, we validated the rationality of the signature using GEPIA (22), HPA (23), cBioPortal (24), TIMER 2.0 (25), STRING (26) and GeneMANIA (27) databases. Specifically, genetic alterations were evaluated in cBioPortal, mRNA and protein expression profiles as well as their prognostic values were evaluated in GEPIA and HPA, levels of immune cell infiltration levels were explored in TIMER 2.0, while protein-protein interaction (PPI) and gene interaction networks were constructed in STRING and GeneMANIA databases.

### Construction of WGCNA Co-Expression Networks and Significant Module Identification

The weight gene co-expression networks of high and low risk-score patients from TCGA-PAAD cohorts were constructed respectively *via* a standard WGCNA procedure (28). A soft power threshold of 9 was selected, based on the criterion of approximate scale-free topology (R^2^ > 0.90), to calculate the adjacencies and Topological Overlap Matrix (TOM) for further clustering gene modules as well as correlating of these modules to risk-scores. The module with a minimum value of preservation Z-summary score was chosen for comparing the co-expression networks between low and high risk-score subgroups and distinguishing them. Moreover, we performed gene ontology (GO) analysis to assess the relevant functional categories of the selected module, then visualized the resulting network *via* Cytoscape software (29). Hub genes and key modules in the network were further identified using cytoHubba and MCODE plugins in Cytoscape.

### Subgroup Analyses of Molecular Characteristics

Several molecular characteristic analyses were performed after assigning TCGA-PAAD and ICGC-PACA-AU patients into high or low risk-score subgroups based on our predictive signature:

1. Gene Set Enrichment Analysis (GSEA) and Gene Set Variation Analysis (GSVA) were conducted, the GO and KEGG gene sets from MSigDB (http://www.gsea-msigdb.org/gsea/msigdb) were chosen as the reference.
2. Cancer-Immunity Cycle, which manages the delicate balance between the recognition of non-self and prevention of autoimmunity, plays an important role in elimination of cancers (30). Expression patterns of genes that inhibited this cycle in training and validation cohorts were explored based on a gene list acquired from Tracking Tumor Immunophenotype website (http://biocc.hrbmu.edu.cn/TIP) (31).
3. The abundance of 22 tumor infiltrating immune cell types were calculated using the Cell‐type Identification By Estimating Relative Subsets Of RNA Transcripts (CIBERSORT) algorithm (32) and presented in violin plots. Another algorithm, the single sample gene set enrichment analysis (ssGSEA) was applied to estimate the immune infiltrations of 16 cell types and 13 immune-associated features (33). The most common immune checkpoint genes, PDCD1 (PD1) and CTLA4 were also assessed.
4. Vésteinn Thorsson et al. identified six immune subtypes across all cancers, namely wound healing (C1), IFN-γ dominant (C2), inflammatory (C3), lymphocyte depleted (C4), immunologically quiet (C5) and TGF-β dominant (C6), which are characterized by differences in macrophage or lymphocyte signatures, Th1:Th2 cell ratio, extent of intratumoral heterogeneity, aneuploidy, extent of neoantigen load, overall cell proliferation, expression of immunomodulatory genes and prognosis respectively (34). We attempted to categorize pancreatic samples in each of the datasets according to this system by “ImmuneSubtypeClassifier” package.
5. Landscapes of somatic mutations and tumor mutation burdens (TMB) of the TCGA-PAAD cohort were visualized using the “maftools” package, while TMB distributions and their influence on OS were explored in the TCGA-PAAD cohort.
6. The Genomics of Drug Sensitivity in Cancer (GDSC) database was explored to predict chemotherapy and targeted therapy responses (35), using statistical models from gene expression (mRNA sequencing data) and drug sensitivity data of cell lines from GDSC. This prediction was performed by the “pRRophetic” package, then half-maximal inhibitory concentrations (IC50s) of each drug were estimated (36). The subclass mapping algorithm was used to predict clinical responses to immune checkpoint blockade by integrating our data with a famous published metastatic melanoma dataset, comprising 47 metastatic melanoma patients that responded to immunotherapies (37, 38). To identify potential compounds targeting high risk-score related biological mode, also known as Mode of Action (MoA), the Broad Institute’s Connectivity Map (CMap) analysis (39) was adopted in microarray datasets, then we selected compounds that were significantly enriched in at least two datasets.

### Histopathological Evaluation

To investigate the corresponding proteins of the four MDGs, we evaluated paraffin-embedded PACA samples obtained from patients subjected to radical surgery from August 2014 to August 2017 in our center. Prior to their inclusion, all patients were required to provide written informed consents according to the International Conference on Harmonization and the Declaration of Helsinki. This retrospective study was approved by the Institutional Review Board (IRB) of Zhujiang Hospital (2021-KY-078-01). To analyze specific marker expressions under consistent conditions, tissue microarrays were constructed for 70 cases of PACA and matched para-cancerous tissues by standard methods (40). The paraffin-coated microarray slides were placed on a 60°C heating block for 20 min and washed with xylene, then incubated in citrate buffer (pH 6.0) for 5 min at 120°C. Endogenous peroxidase was blocked by 0.3% H_2_O_2_ for 10 min. The slides were incubated with 5% BSA in PBS at room temperature for 1 h, followed by incubation with appropriated primary antibodies at 4°C for overnight, then with horseradish peroxidase (HRP) anti-rabbit IgG antibodies for 1 h. Then color was developed by incubation with DAB Substrate kit (ZSGB-BIO, ZLI-9017). After washing in PBS, tissue sections were counterstained with hematoxylin. The primary antibodies used in our study included anti-human GPRC5A antibody (1:200, ABclonal, A8173), anti-human SOWAHC antibody (1:300, Proteintech, 24033-1-AP), anti-human S100A14 antibody (1:300, ABclonal, A10394), and anti-human ARNTL2 antibody (1:150, Bioss, BS-11446R). Five representative fields of each sample (at least three for extremely fibrotic or necrotic samples) were selected using CaseViewer 2.4 software to ensure homogeneity as well as representativeness at a magnification of 400 X. Expression levels of the four MDGs were evaluated as average optical density (AOD) using ImageJ software (41, 42). Experimental data were statistically analyzed by paired t-test. The associations between protein expression levels and OS/RFS of patients were explored by Kaplan–Meier survival analyses and Cox regression analyses.

### Statistical Analysis

All data-mining work was performed using packages, limma (Version 3.46.0) (43), MethylMix (Version 2.20.0) (18), clusterProfiler (Version 4.3.1) (44), survminer (Version 0.4.9), survival (Version 3.2.13), glmnet (Version 4.1.2) (45), pheatmap (Version 1.0.12), corrplot (Version 0.90), timeROC (Version 0.4) (46), WGCNA (Version 1.70.3) (28), maftools (Version 2.6.5) (47) and pRRophetic (Version 0.5) implemented in R software, version 4.0.2. For all analyses, two-tailed p ≤ 0.05 was set as the threshold for determining statistical significance.

## Results

### Differentially Expressed Methylation-Driven Genes

**Figure 2:**
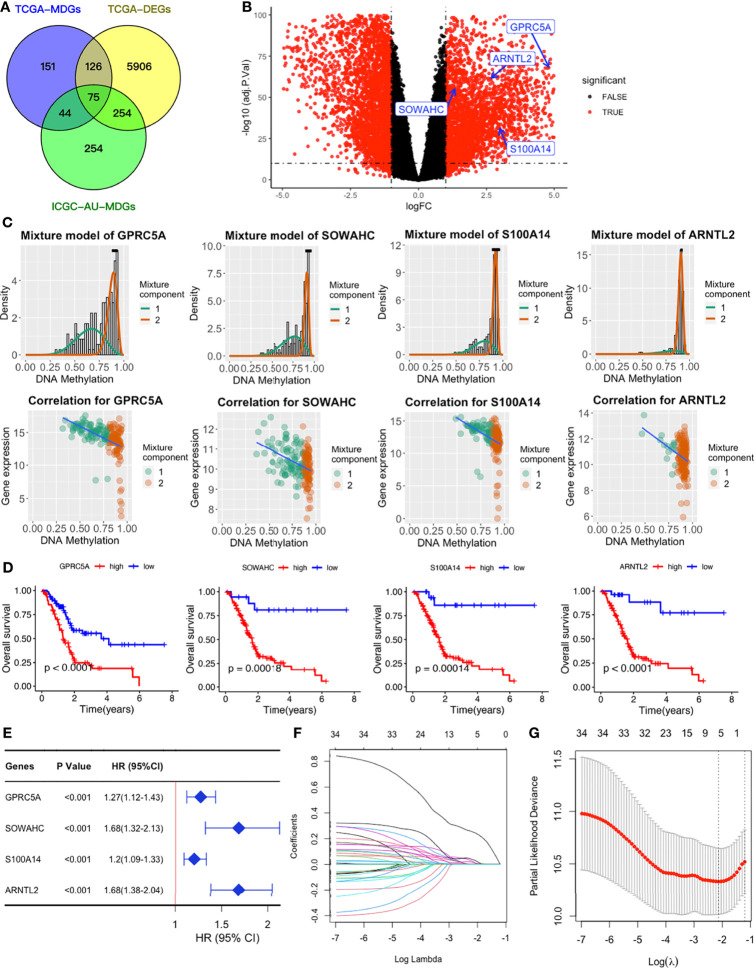
Establishment of a four-MDG signature. **(A)** Venn diagram showing DEGs and MDGs from the TCGA and ICGC datasets. **(B)** Volcano plot of DEGs with the four-MDG signature gene marked. **(C)** Distribution maps showing the degree of methylation degree (the top row) of the four signature genes and their correlation plots (the bottom row) between mRNA expression and DNAm levels. **(D)** K-M curves for the four MDGs in the TCGA dataset. **(E)** Univariate Cox regression of the four signature genes in the TCGA dataset. **(F)** LASSO coefficient profiles of the 35 genes in the TCGA dataset. **(G)** Selection of the optimal parameter (lambda) in the LASSO model.

We obtained a total of 350 samples (179 tumor and 171 normal) with 183 and 167 samples being obtained from TCGA and GTEx databases, respectively. RNA sequence, DNAm, and clinical data were available for 182 samples. Analysis of gene expression and DNAm data using MethyMix algorithm and thresholds of |log2FC| > 1, p < 0.05 and Cor < -0.3 as cut-offs, resulted in identification of 6361 DEGs and 75 differentially expressed MDGs. Among the MDGs, 53 were found to be hypomethylated and elevated in tumor samples while 22 were hypermethylated and down-regulated (**Figures 2A–C** and **Table S1**). Functional enrichment analysis revealed that up-regulated DEGs were significantly enriched (p < 0.05) in terms related to tumor microenvironments and immune texture, including collagen formation, degradation of extracellular matrix organization and rheumatoid arthritis (**Figure S1C**). Meanwhile, up-regulated MDGs were enriched in the IL-3 signaling pathway, FGFR1 as well as S1P3 pathways and some important biological processes, including cell adhesion, epithelial to mesenchymal transition (**Figure S1E**). Down-regulated DEGs and MDGs were enriched in pathways regulating normal functions, including glucagon signaling and pancreatic secretion (**Figures S1D, F**).

### Construction of MDG-Based Signature

**Figure 3:**
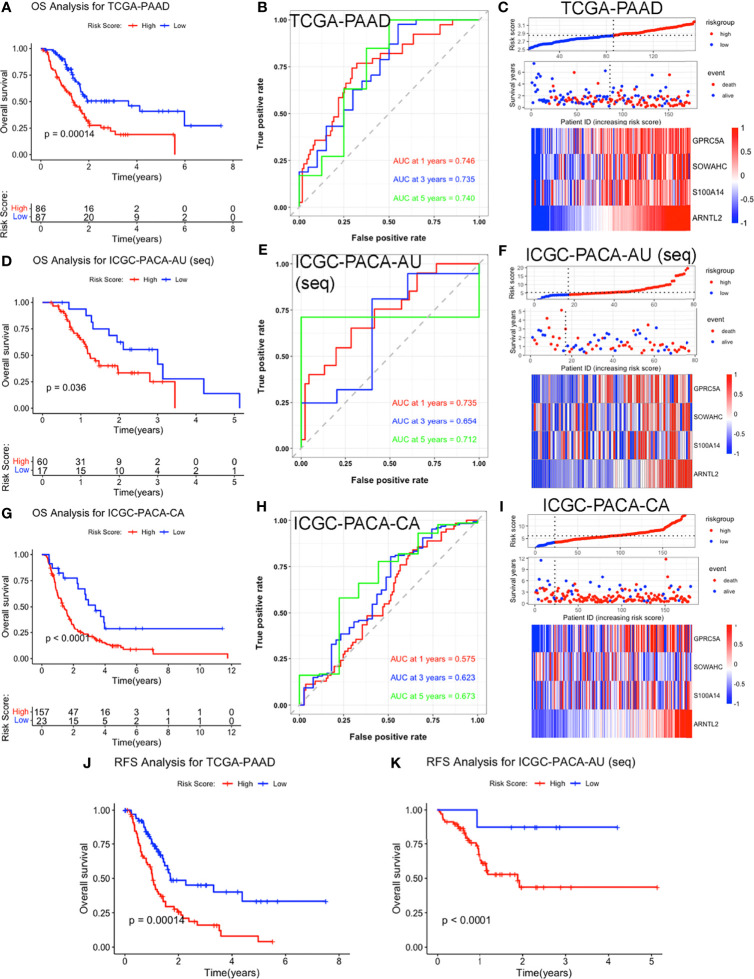
Validation of the four-MDG signature in the TCGA-PAAD, ICGC-PACA-AU (seq), and ICGC-PACA-CA datasets. **(A, D, G)** K-M survival curves showing OS of patients in the high and low risk-score subgroups across three datasets. **(B, E, H)** Time‐dependent ROC curves of the signature. **(C, F, I)** Risk-score distribution plots for the three datasets. In each plot, from top to bottom: distribution of risk-scores, distribution of survival status, expression patterns of the four genes. **(J, K)** K-M analysis for RFS of high and low risk-score subgroups in TCGA-PAAD and ICGC-PACA-AU (seq) datasets.

To assess their relationships with OS, the 75 differentially expressed MDGs were subjected to K-M survival and univariate Cox regression analyses. Among them, 35 MDGs were significantly correlated with OS (p < 0.05) (**Table 1** and **Figures 2D, E****)**. To further shrink the gene screening scope, these candidate MDGs featured coefficients (not zero) in a further LASSO multivariate Cox regression model, in which these genes were required to appear 1000 times of 1000 repetitions. The penalty was established through 10-fold cross‐validations (**Figures 2F, G****)**. Finally, four MDGs, GPRC5A, SOWAHC, S100A14 and ARNTL2, were selected as prognostic genes for the signature. Notably, methylation was inversely correlated with mRNA expression levels of these signature genes (**Figure 2C**). Then, the predictive model was established by adding the product of the expression level and relative coefficient of each gene in the LASSO regression as follows: Risk score = (0.009380 * expression value of GPRC5A) + (0.014534 * SOWAHC expression value) + (0.002176 * S100A14 expression value) + (0.227490 * ARNTL2 expression value). Positive coefficients of these genes implied that their upregulation represented poor OS for PACA patients. Therefore, patients in the high risk-core subgroup exhibited significantly worse OS than those in the low-risk subgroup, while AUCs of ROC for 1-, 3-, and 5-year OS were 0.746, 0.735 and 0.740, respectively. Overall, these results indicated that our model had a high predictive value (**Figures 3A–C**).

**Table 1:** Univariate Cox regression analysis results of the 35 MDGs.

Gene	HR	HR (95% CI)	P value
KLF4	1.26	1.26 (1.06–1.48)	0.007
ARL4D	1.24	1.24 (1.05–1.47)	0.012
GPRC5A	1.27	1.27 (1.12–1.43)	<0.001
DOK5	1.2	1.2 (1.05–1.38)	0.01
EPHX4	1.28	1.28 (1.11–1.47)	<0.001
ANXA13	1.11	1.11 (1.01–1.21)	0.027
SLC45A3	1.22	1.22 (1.06–1.4)	0.004
PTPRH	1.2	1.2 (1.03–1.4)	0.019
SEC11C	0.77	0.77 (0.6–0.98)	0.035
LRRC31	1.08	1.08 (1.01–1.17)	0.033
CXCL3	1.12	1.12 (1.02–1.23)	0.022
PHYHD1	1.24	1.24 (1.06–1.46)	0.008
CSTA	1.18	1.18 (1.05–1.32)	0.005
IGFL3	1.17	1.17 (1.08–1.27)	<0.001
HCAR1	1.13	1.13 (1.03–1.24)	0.01
TMEM97	1.28	1.28 (1.1–1.5)	0.002
CLDN18	1.05	1.05 (1–1.11)	0.036
AMIGO2	1.45	1.45 (1.22–1.73)	<0.001
SOWAHC	1.68	1.68 (1.32–2.13)	<0.001
CLDN23	1.16	1.16 (1.02–1.31)	0.023
HTR1B	1.12	1.12 (1.01–1.24)	0.039
MTMR11	1.19	1.19 (1.02–1.38)	0.031
CLDN4	1.26	1.26 (1.09–1.47)	0.003
S100P	1.17	1.17 (1.07–1.27)	<0.001
FXYD3	1.19	1.19 (1.04–1.36)	0.012
PPP1R14D	1.19	1.19 (1.07–1.33)	0.001
LPCAT4	1.26	1.26 (1.08–1.47)	0.004
KRT20	1.06	1.06 (1.01–1.11)	0.026
AGR2	1.11	1.11 (1.02–1.22)	0.023
S100A14	1.2	1.2 (1.09–1.33)	<0.001
ARNTL2	1.68	1.68 (1.38–2.04)	<0.001
C5orf38	0.89	0.89 (0.81–0.97)	0.009
GREB1L	1.17	1.17 (1.04–1.32)	0.01
CD55	1.22	1.22 (1.05–1.41)	0.01
S100A2	1.16	1.16 (1.08–1.25)	<0.001

### Validation of the Four-MDG Signature

#### Survival Analysis

We validated the established signature using ICGC, GEO and ArrayExpress datasets. Specifically, survival analyses showed that high risk scores were significant predictors for OS. Meanwhile, AUCs for 1-, 3-, and 5-year OS in these validation datasets were all larger than 0.5 in validation datasets, indicating the robustness of the predictive value of our four-MDG signature (**Figures 3A–I** and **Figure S2A–I**). Although our signature was constructed based on OS data of the TCGA-PAAD cohort, K-M curves for recurrence-free survival (RFS) revealed a positive correlation between higher risk-scores and shorter RFS in PACA patients (**Figures 3J, K** and **Figure S2J, K**).

#### Univariate and Multivariate Cox Regression Analysis

To investigate whether our four-MDG signature was an independent prognostic biomarker, we performed Cox regression analysis in the TCGA-PAAD dataset (**Figure S3**). Both univariate and multivariate analyses revealed that the risk-score was a positive risk factor for OS of PACA patients. Hazard ratio (HR) values and their corresponding 95% confidence interval (95% CI) were 7.542 (3.238-17.567) and 7.489(3.063-18.311) for univariate and multivariate analyses, respectively. In the validation datasets, ICGC-PACA-AU (seq), ICGC-PACA-AU (array) and E-MTAB-6134 datasets, the signature also showed the potential to work as an independent prognostic marker (**Figure S3**).

#### Correlations With Clinical Features

1We investigated whether some important clinicopathological features, namely histologic grades (G grades, G1: well differentiated, G2: moderately differentiated, G3: poorly differentiated, G4: undifferentiated) (48, 49) and TNM stages, which have long been ascertained to be prognostic in PACA patients (50, 51), were correlated with our signature. In all the four datasets TCGA-PAAD, E-MTAB-6134, GSE62452 and GSE78229 providing G grade data, highly graded samples tended to exhibit higher risk-scores (**Figure S4A**), indicating that higher risk-scores may be associated with lower differentiated and more malignant tumors.

In the TCGA dataset, risk scores were remarkably different among signature-stratified patients, with higher risk scores in T2 stage and Stage 1 patients. Moreover, it seemed that older patients recorded higher risk scores than their young counterparts although the correlation between age and risk-scores was not statistically significant (**Figure S4B**). In other datasets, the age/risk-score correlations were either non-significant (**Figure S4C**) or unavailable due to lack of the necessary data. These results suggested that independent of gender and age, our four-MDG signature can stratify patients with different pathological stages.

#### Characteristic of Each Signature Gene

We analyzed the expressions of 4 signature genes, GPRC5A, SOWAHC, S100A14 and ARNTL2 using online tools, and found that their mRNA values were all upregulated in PACA, relative to adjacent normal tissues (**Figure S5B**). These high expressions not only indicated shorter OS, but were also negatively correlated with disease free survival (DFS) (**Figure S5A**). Moreover, immunohistochemical pathology (IHC) revealed that corresponding proteins encoded by GPRC5A, SOWAHC, and ARNTL2 genes predicted unfavorable prognosis (**Figure S5C**). Apart from being affected by dysregulated methylation, these upregulated MDGs could also be concurrently influenced by gene amplifications, a main type of copy number variation (CNV). Screening the cBioPortal website revealed that their gene amplifications were detected in 0 - 4% of TCGA-PAAD patients (**Figure S5D**), indicating that they have minimal influence on gene expressions. PPI and gene-gene networks obtained from STRING and GENEMANIA databases revealed that these four genes do not closely interact with each other (**Figures S6A, B**).

We performed further analyses using TIMER 2.0, a comprehensive website for systematical analysis of immune infiltrates, while all analyses were adjusted by tumor purity and only the Pearson correlation coefficients were obtained. We adopted CIBERSORT, the most common and accurate algorithm for immune infiltration estimating, for analysis of all cell types except for myeloid-derived suppressor cells (MDSCs), whose abundance was obtained by Tumor Immune Dysfunction and Exclusion (TIDE) algorithm (52). All statistically significant correlations are shown in **Figure S7**. In summary, signature genes were negatively correlated with CD8+ T cells, but positively with immunosuppressive cell types, such as T regulatory cells (Tregs), M0 macrophages, dendritic cells (DCs) and MDSCs.

### Methylation Status of the Signature Genes

**Figure 4:**
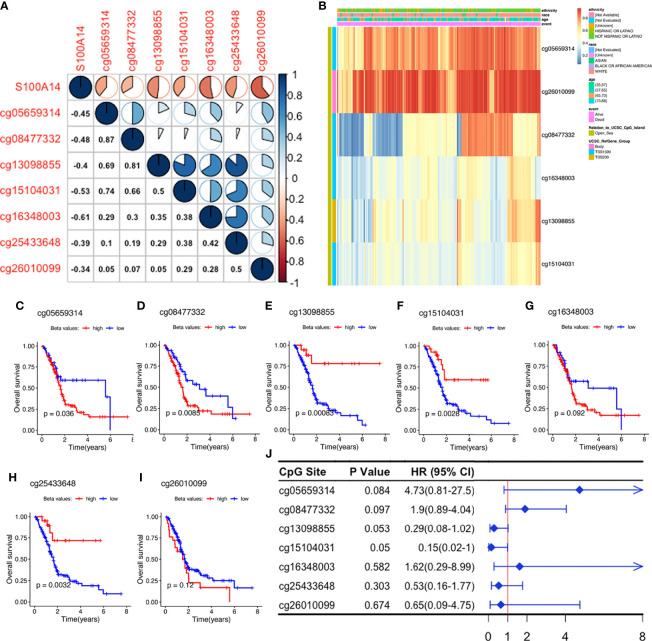
Exploration of S100A14 cg sites. **(A)** Pearson’s correlation between DNAm levels of the cg sites and S100A14 mRNA expression levels. **(B)** DNAm beta value heatmaps of S100A14. **(C–I)** K-M survival curves showing OS of patients with high and low beta values at each cg site in the TCGA-PAAD dataset. **(J)** Univariate Cox regression analysis for OS of S100A14 cg sites in the TCGA dataset.

Correlation heatmaps showed that S100A14 expression was inversely associated with methylation levels across all the seven CpG sites, although their beta values revealed different coefficients with S100A14 expression (**Figures 4A, B****)**. We further analyzed the relationship between methylation levels (beta values) and patient outcomes, and found that beta values of 3/7 S100A14 cg sites were significantly correlated with shorter OS, while another 3/7 were significantly associated with better outcomes (**Figures 4C–J**). Comparable findings were observed in cg sites of GPRC5A, SOWAHC and ARNTL2 genes (**Table S2** and **Figures S8A–C**). These findings showed that DNAm and expression levels, as well as their influence on OS, were not consistent. Particularly, not all CpG sites exhibited significant correlations with expressions of the corresponding genes or patient prognosis (**Figure 4J**), indicating the intricate influence of epigenetic regulation. Due to the complexity of CpG deviations, MDGs might be more suitable to serve as biomarker candidates for PACA, reflecting both epigenetic and functional (or transcriptomic) situations.

### Co-Expression Network Construction and Module Identification

**Figure 5:**
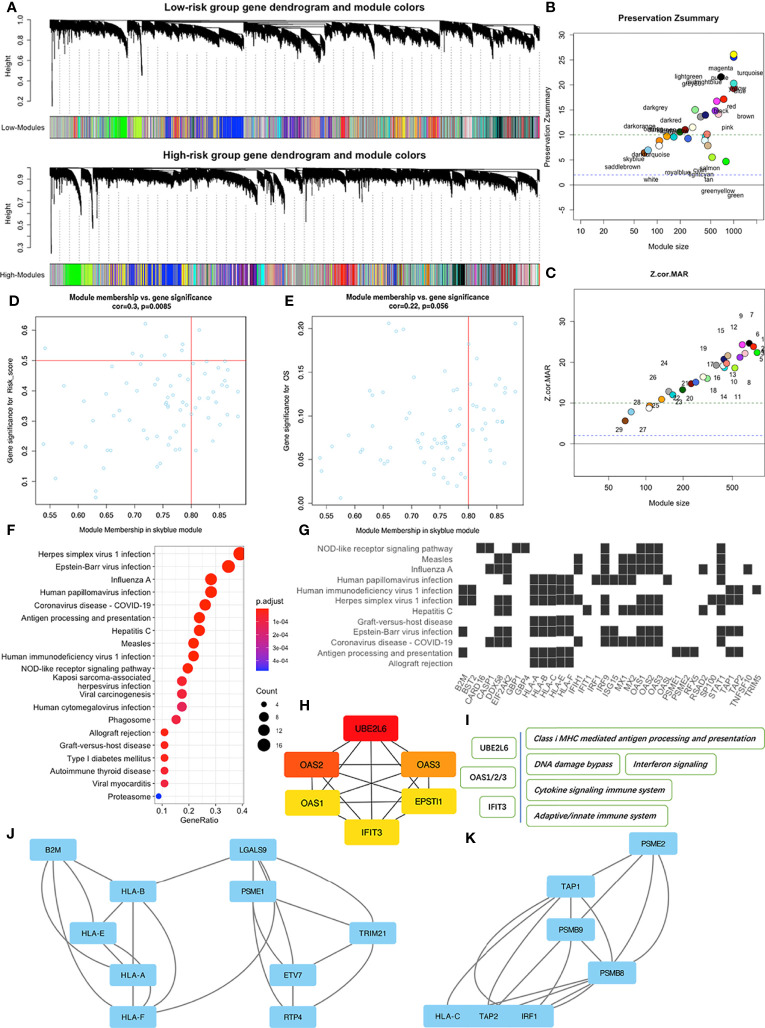
WGCNA of patients in the high and low risk-score subgroups in the TCGA-PAAD dataset. **(A)** Dendrograms of high and low risk-score subgroups. **(B)** The Preservation Zsummary of co-expression modules. **(C)** The Preservation Z-Statistics of co-expression modules. **(D)** Scatter plot for the module membership vs. gene significance for risk-scores in the skyblue module. **(E)** Scatter plot for module membership vs. gene significance for OS in the skyblue module. **(F)** Functional enrichment analysis of skyblue module genes for KEGG pathways. **(G)** Distribution of some skyblue module genes involved in specific links in the enriched KEGG pathways. **(H)** The six hub genes of skyblue module identified by cytoHubba. **(I)** Some pathways associated with the hub genes. **(J, K)** Two of the three key sub networks of the skyblue module.

During WGCNA analysis, we constructed two co-expression networks for high or low risk-score patients and clustered genes using average-linkage hierarchical clustering. Results were segmented according to the set criteria to obtain different gene modules, then two clustering trees (dendrograms) with 30 modules were plotted for each subgroup (**Figure 5A**). Although we found no perfect consistency between two dendrograms, we hypothesized that most of the modules would show significant preservation between the subgroups, and that those non-preserved modules could explain the changes of network properties between the high and low risk-score subgroup networks. Results indicated that most modules showed strong evidence of preservation, with the exception of skyblue and saddlebrown, which contain 76 and 68 genes respectively (**Figures 5B, C****)**. However, further enrichment analysis revealed that saddlebrown module genes were not enriched in any pathway. Therefore, we chose skyblue as the non-preserved module. The low Z-summary score obtained in the skyblue module implied a low degree of preservation, indicating that it was possible to distinguish expression levels of module genes between patients with high and low risk- scores (**Figure 5D**). However, this module seemed to be not significantly related to OS (**Figure 5E**) since the p value was just above 0.05.

To identify features associated with our four-MDG signature, we performed a more detailed analysis of the skyblue module. Results from GO enrichment analysis (**Figure 5F**) showed that the 76 module genes were mainly enriched in two main biological processes, namely immune dysregulations (comprising Antigen processing and presentation, Allograft rejection, and Autoimmune thyroid disease, among others) and virus infection reactions (such as EB virus infection, Hepatitis C infection, and Viral myocarditis). A detailed description of the specific link between those enriched genes and corresponding biological processes is shown in **Figure 5G**. To identify key nodes in the module, we adopted Cytoscape software and its plugin cytoHubba to calculate the strength of intra-module connectivity of each gene. The top six hub genes identified included UBE2L6, OAS1, OAS2, OAS3, EPSTI1 and IFIT3, most of which were associated with immune regulation and response to DNA damage, according to the GeneCards database (https://www.genecards.org/) (**Figures 5H, I****)**. The MCODE plugin revealed the key sub-networks of the skyblue module (**Figures 5J, K****)**. The module’s nodes included some HLA genes, suggesting that these networks could be involved in immune responses especially antigen presenting. Overall, these results suggested that the four-MDG signature may be associated with tumor immune functions.

### Functional Annotation of the Signature

**Figure 6:**
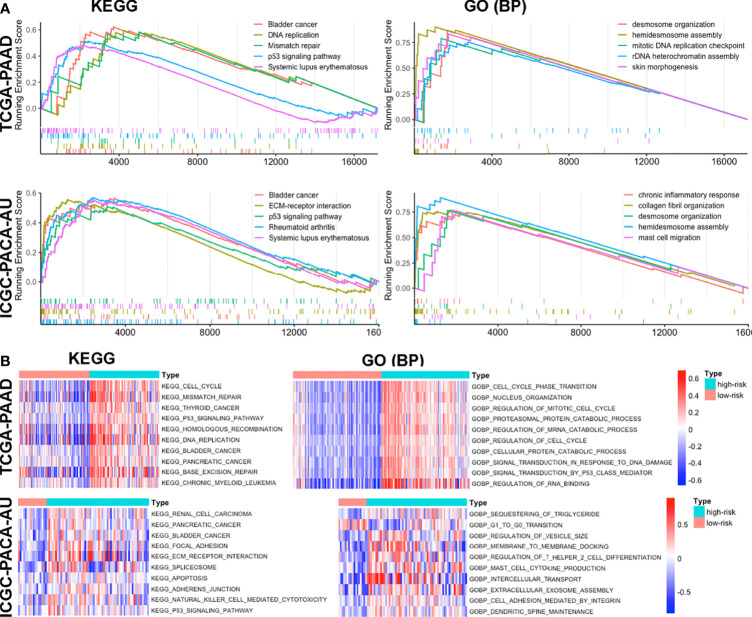
Functional enrichment of the signature based on GSEA **(A)** and GSVA **(B)** in TCGA-PAAD and ICGC-PACA-AU datasets.

To further elucidate the possible mechanisms underlying our signature model, we performed GSEA to identify enriched KEGG pathways and GO biological processes in TCGA-PAAD, ICGC-PACA-AU (seq), ICGC-PACA-CA and E-MTAB-6134 data sets. The top 50 GO biological processes and KEGG pathways were selected according to logFC values in each dataset (**Table S3**). Some pathways and processes, such as p53 signaling pathway, mismatch repair, bladder cancer and systemic lupus erythematosus, were repeatedly enriched in high risk-score subgroups, indicating that higher risk-scores were associated with carcinogenesis, immune dysregulation, mismatch repair and DNA damage responses (**Figure 6A** and **Figure S9A**). Furthermore, we performed GSVA to analyze differentially enriched pathways. Results revealed significant enrichment of the p53 signaling pathway, mismatch repair, carcinogenesis of several malignancies, as well as ERBB and VEGF signaling pathways (**Figure 6B**, **Figure S9B** and **Table S4**). Overall, these results indicated that high risk-scores of the signature were associated with activation of the tumor suppressor p53, DNA mismatch repair, disturbed immune system and cancer intrinsic pathways.

### High Signature Risk-Scores Indicate Immunosuppressive Features of Tumor Microenvironment

#### Immune Infiltrations and Checkpoint Gene Expressions

Based on the variation in immune-related pathways or processes enriched among subgroups, we evaluated immune infiltration levels to further characterize their immunologic landscapes across TCGA-PAAD, ICGC-PACA-AU and E-MTAB-6134 datasets.

**Figure 7:**
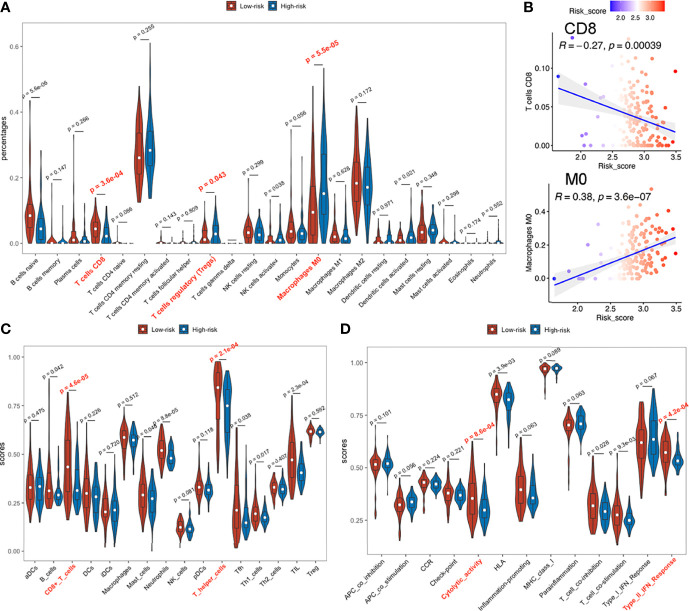
Immune infiltration status of TCGA-PAAD dataset. **(A)** Abundances of 22 immune cells by CIBERSORT. **(B)** Upper: correlation between of CD8+ T cell infiltration with the signature; lower: correlation between M0 macrophage with the signature. **(C, D)** Immune cell infiltration analysis and immune function enrichment by ssGSEA.

Patients in high and low risk scores subgroups exhibited significant differences, with higher abundance of CD8+ T cells, lower infiltrations of regulatory T (Treg) cells and M0 macrophages (naive or non-polarized macrophages) observed in low risk-score subgroups during CIBERSORT analysis (**Figure 7A**) in TCGA-PAAD and E-MTAB-6134 datasets (**Figure S12A**), suggesting a possible association between our established signature and the immunosuppressive microenvironment. ICGC-PACA-AU dataset showed a remarkable trend that lower risk-score samples were more infiltrated with CD8+ T cells, although did not reach statistical significance (**Figures S10A**, **S11A**). Correlation plots demonstrated that risk-scores were negatively associated with infiltration of CD8+ T cells, but positively correlated with M0 macrophages (**Figure 7B**). In ssGSEA analysis, 2 immune cell types (CD8+ T cells and T helper cells) and 2 immune functions (Cytolytic activity and Type II IFN responses) were significantly related to risk-scores in all datasets (**Figures 7C, D** and **Figure S10**–**12C, D**).

In TCGA-PAAD and E-MTAB-6134 datasets, PD1 and CTLA4 expressions were significantly higher in low risk-score, than in high risk-score subgroups (**Figure S13A, B**), while differences in the ICGC-PACA-AU datasets were not statistically significant. Since PD1 and CTLA4 are mainly expressed on the surface of lymphocytes, their upregulation may have resulted from the abundantly infiltrated immune cells. Notably, elevated PD1 or CTLA4 levels indicate an exhausted status of immune cells, which is a common occurrence in cancers (53).

#### Negative Regulatory Genes of the Cancer-Immunity Cycle

**Figure 8:**
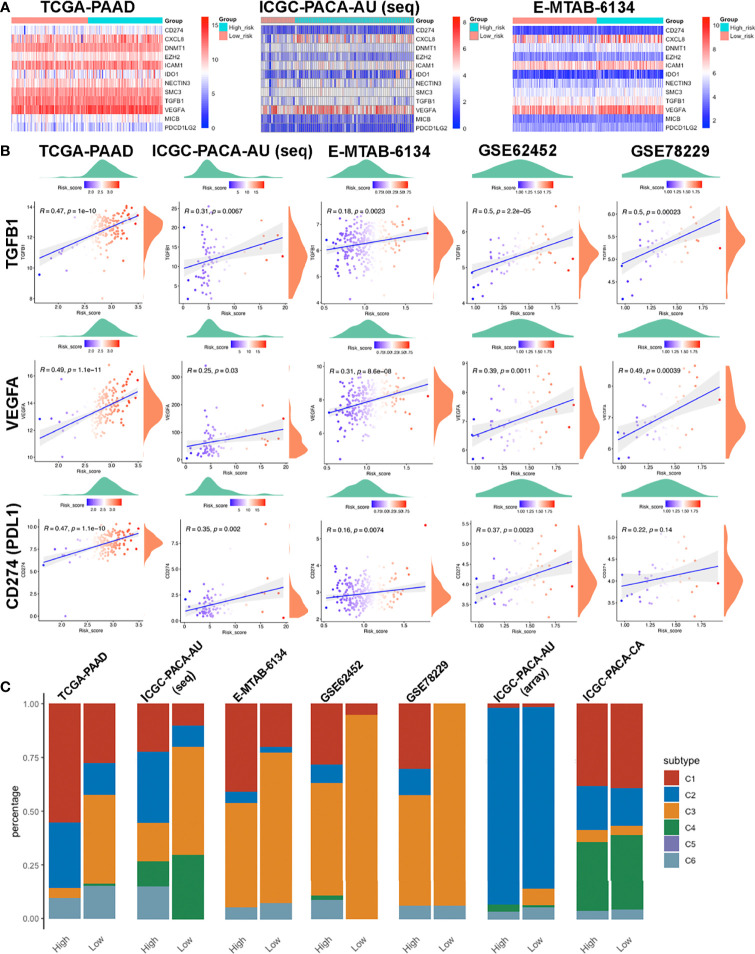
Features of the immune-related biomarkers between patients in high and low risk-score subgroups. **(A)** Heatmaps of 12 CIC negative regulatory genes in TCGA-PAAD, ICGC-PACA-AU and E-MTAB-6134 datasets. **(B)** Correlations between CTLA4, PDCD1, CD274 (PDL1) and risk-scores in TCGA-PAAD, ICGC-PACA-AU (seq), E-MTAB-6134, GSE62452 and GSE78229 datasets. **(C)** Distribution of immune subtypes across all included datasets.

We downloaded the 42 negative regulatory cancer-immunity cycle (CIC) genes from the TIP website, then analyzed their expression patterns across each cohort. Expression levels for twelve of them, namely CD274, CXCL8, DNMT1, EZH2, ICAM1, IDO1, NECTIN3, SMC3, TGFB1, VEGFA, MICB, and PDCD1LG2, were positively correlated with risk scores, in at least four datasets, with no negative correlation in any dataset. Most of these genes were upregulated in the high-risk group (**Figures 8A, B****)**. Among them, TGFB1 and VEGFA are tumor-secreted immunosuppressive factors, while CD274 (PDL1) and PDCD1LG2 are both PD1 ligands and cell-surface immunosuppressive factors. Notably, elevated PDL1 levels have been associated with poor prognostic outcomes (54, 55).

#### Immune Subtypes

To further investigate the characteristics of the immune microenvironment in PACA tissues, we categorized samples in each datasets. TCGA-PAAD, ICGC-PACA-AU (seq), E-MTAB-6134, GSE62452 and GSE78229 datasets exhibited similar patterns, while ICGC-PACA-AU (array) and ICGC-PACA-CA showed differently. This was attributed to possible heterogeneities across different studies and platforms. Notably, in almost all the datasets, C3 (inflammatory) subtypes were more abundant in the low risk-score subgroup, than in high risk-score subgroups. Moreover, C3 and C2 (IFN-γ dominant) subtypes were predominant in most low risk groups (**Figure 8C**). Previous studies have shown that C3 subtypes are associated with the most favorable prognosis, due to the type I immune response needed for cancer control (56) as well as the most pronounced Th17 signature (57), C2 was IFN-γ dominant and showed a less favorable survival outcome compared to C3 (34).

### Somatic Mutation Landscape of the Subgroups

**Figure 9:**
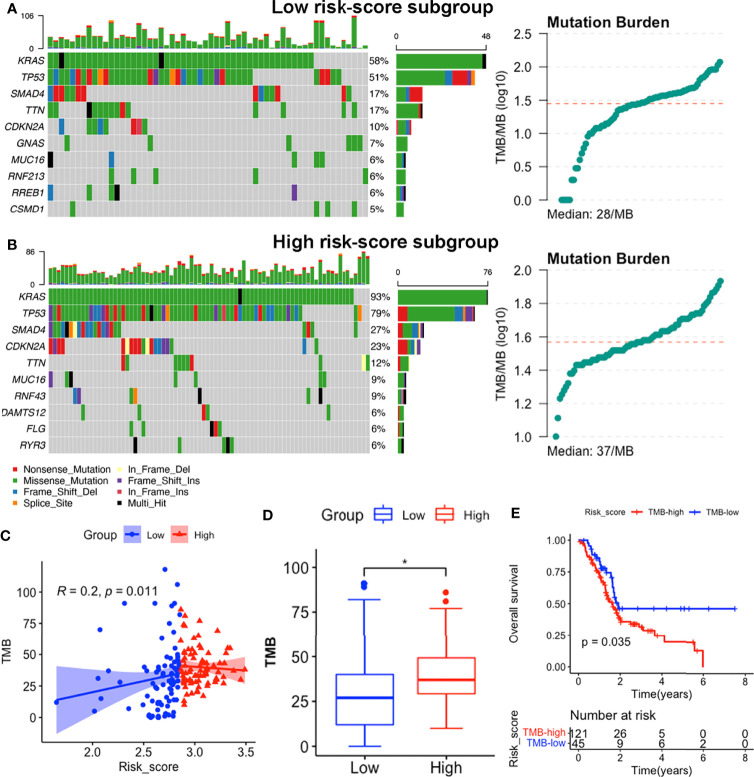
Somatic mutation features between subgroups in the TCGA-PAAD dataset. **(A, B)** Somatic mutation landscape and TMB status of low-risk **(A)** and high-risk **(B)** subgroups. **(C)** Correlation between TMB levels with risk-scores. **(D)** Comparisons of TMB distributions of the two subgroups by t-test. **(E)** K-M survival curves showing OS of patients with high and low TMB. (*p<0.05).

We identified the top 10 genes with the highest mutation rates in each subgroup (**Figures 9A, B****)**. Notably, KRAS, TP53, SMAD4 and CDKN2A mutations were the most common in both subgroups, of which KRAS and TP53 were the most dominant, with rates above 50% in both groups. Missense mutations were the most common, followed by nonsense and frameshift deletions. Moreover, patients in the high risk-score subgroup (median TMB: 37mut/Mb) exhibited higher TMBs than those in the low risk-score subgroup (median TMB: 28mut/Mb, p < 0.05, **Figures 9A, B, D****)**, implying that patients with high-risk scores have better responses to immune checkpoint blockade (ICB) treatment (58). Notably, signature risk-scores were positively correlated with TMB (R = 0.2, p = 0.011, **Figure 9C**), while survival analyses showed that higher TMB levels were significantly associated with shorter OS (p = 0.035, **Figure 9E**).

### Prediction of Therapeutic Responses

**Figure 10:**
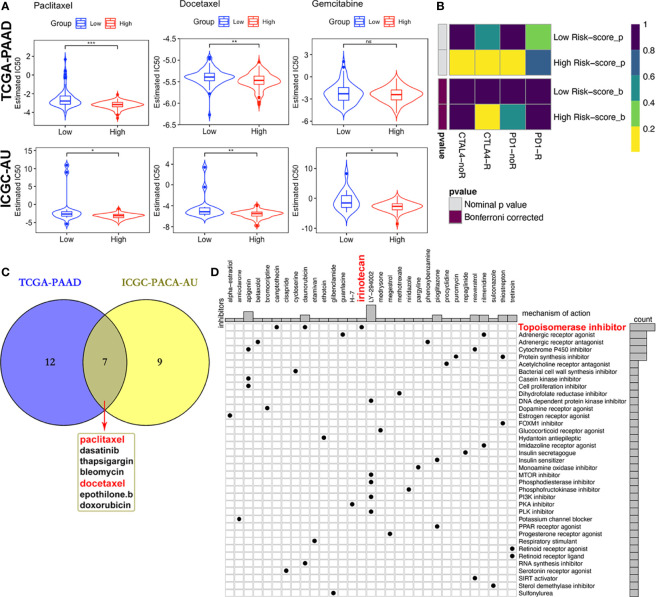
Differential putative therapeutic responses. **(A)** Boxplots showing estimated IC50 values for docetaxel, paclitaxel and gemcitabine in the TCGA-PAAD and ICGC-PACA-AU datasets. **(B)** Submap analysis revealed that patients with high scores might be more sensitive to CTLA-4 blockade therapy (Bonferroni-corrected P = 0.02). **(C)** Venn plot of candidate chemo and targeted drugs identified by the GDSC database in TCGA and ICGC-AU datasets. **(D)** CMap database analysis identified potential compounds targeting the four-MDG signature. (*p<0.05, **p<0.01, ***p<0.001, ns, non-significant).

Due to differences in TMB between subgroups, we investigated the likelihood of responses to immune checkpoint blockade (ICB) therapy. Currently, the ICB therapy has not yet been approved as a routine treatment strategy for PACA patients, while stratification biomarkers are still debatable. We adopted the Subclass mapping algorithm to compare the expression profiles of the two subtypes we defined in the previously mentioned dataset (38). to those of the TCGA-PAAD dataset and predicted the likelihood of immunotherapeutic responses. Patients in the high-risk subgroup were found to be more likely to respond to CTLA-4 ICB therapy than those with low risk-scores (Bonferroni corrected p* *= 0.02, **Figure 10B**).

Given that chemotherapies, such as FOLFIRINOX (5-fluorouracil, leucovorin, oxaliplatin and irinotecan) and gemcitabine plus nab-paclitaxel regimens, remain the standard strategies for the clinical management of advanced PACA (59), with targeted therapy being of benefit to a small set of patients, we tried to assess responses to drugs collected in GDSC database by integrating and analyzing the transcriptomic data of TCGA-PAAD and ICGC-PACA-AU (seq) cohorts with those data embedded in GDSC. Results showed that some of these drugs exhibited significantly lower IC50 values, indicative of more sensitivity in one subgroup relative to the other (**Figure 10C** and **Table S5**). Notably, patients in the high risk-score subgroup in both cohorts were found to be more sensitive to three chemotherapy drugs (docetaxel, paclitaxel and gemcitabine), although differences of gemcitabine IC50 distribution subjects in the TCGA cohort were non-significant (**Figure 10A**). Besides, high risk score patients in both cohorts were sensitive to dasatinib and bleomycin (**Figure S14**).

Moreover, MoA analysis revealed 31 compounds targeting 37 biological actions or pathways, including the topoisomerase irinotecan, which is one of the basic components of the FOLFIRINOX regimen (**Table S6** and **Figure 10C**).

### Histopathologic Validation of the Four MDGs

We validated the prognostic values of the MDG expressions *via* TMA-based IHC experiments in 70 paired PACA samples and adjacent non-tumorous tissues. Due to high heterogenous and fibrous features of PACA, 18 ineffective pairs were eliminated. The elimination criteria were: (1) Fibrous cancerous tissue with less than three representative fields containing more than 50 cells, (2) Adjacent tissues containing typical malignant cells, and (3) Tumorous tissues full of mucous deposit. The clinicopathological characteristics of PACA patients were shown in **Table 2** and **Table S7**.

**Table 2:** Datasets included in this study.

	TCGA-PAAD	ICGC-PACA-AU	ICGC-PACA-CA	GSE62452	GSE78229	E-MTAB-6134	GTEx	Our center
**Country**	USA	Australia	USA	Germany	Germany	France, Belgium	Europe	China
**Sample size (normal/tumor)**	4/179	0/430	0/314	61/69	0/50	0/309	167/0	52/52
**Age (mean [min, max])**	64.9 [35, 88]	66.4 [33, 90]	65.2 [34, 88]	69 [46, 87]	65.1 (SD 9.8)*	64 [36, 87]	–	58.8 [35, 75]
**Gender (Male/Female)**	97/80	236/193	152/118	–	–	179/130	–	31/21
**TNM**								
**T (1/2/3/4)**	7/21/140/3	-/3/4/-	-/2/16/-	–	–	12/45/252/0	–	6/22/23/1
**N (0/1)**	48/125	5/-	10/19	–	–	78/231	–	31/21
**M (0/1)**	168/5	–	7/2	–	–	–	–	52/0
**Stage (I/II/III/IV)**	19/143/3/5	3/-/-/-	52/101/9/1	4/45/13/6	4/45/1/0	–	–	17/29/6/0
**Histologic Stage (G 1/2/3/4)**	24/94/47/4	–	–	2/35/30/2	2/24/22/2	110/130/48/0	–	–
**Survival data**	OS, RFS	OS, RFS	OS, RFS	OS	OS	OS, RFS	–	OS, RFS
**Platform**	Illumina RNAseq	Illumina RNAseq	Illumina RNAseq	Affymetrix Array	Affymetrix Array	Affymetrix Array	Illumina RNAseq	IHC

*SD, standard deviation.

**Figure 11:**
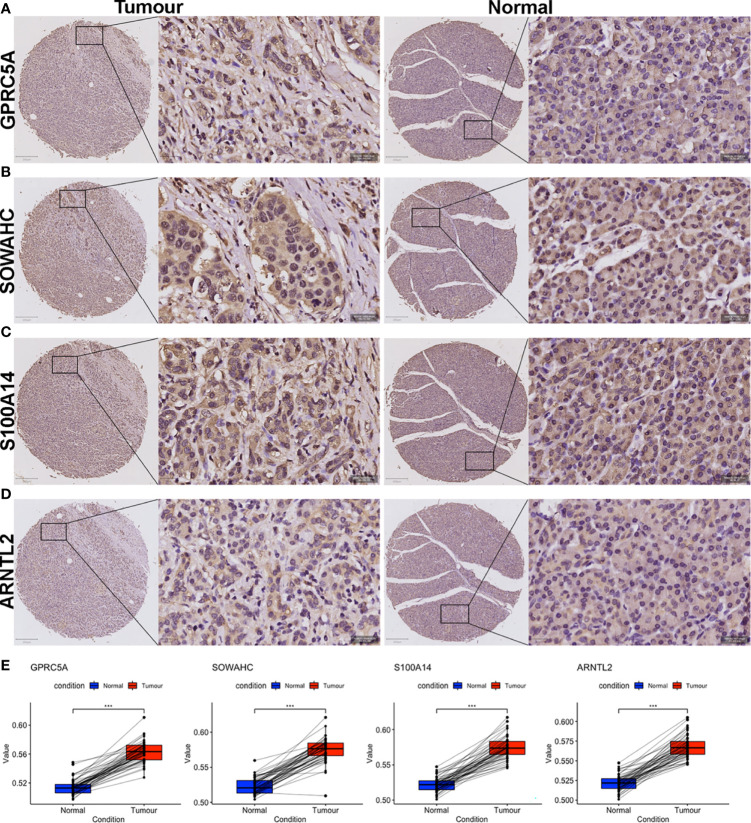
IHC evaluation of PACA samples collected from our center. **(A–H)** Expressions of the four MDG proteins in tumorous and corresponding para-tumor tissues. **(I)** The MDG-corresponding protein levels were up-regulated in PACA tissues compared to non-cancerous tissues. (***p<0.001).

**Figure 12:**
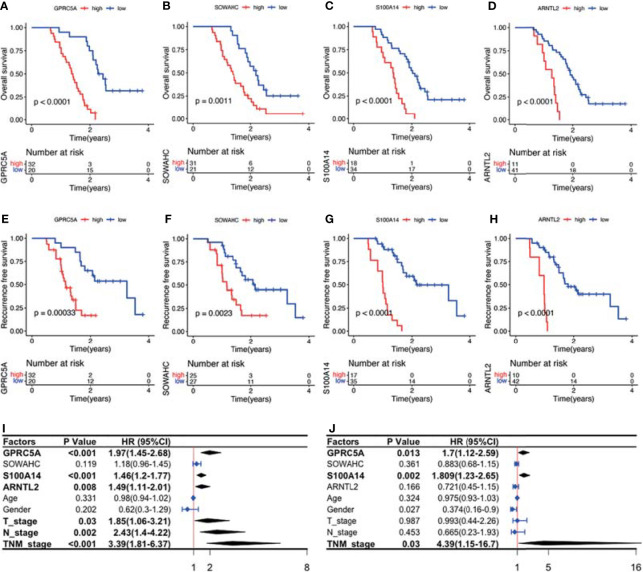
Survival analysis and Cox regression analyses of the association between the four MDG protein levels and patient prognosis. **(A–H)** The MDG protein expression levels were associated with shorter OS and RFS. **(I, J)** Uni- and multi- variate cox regression analyses of the clinicopathological factors of PACA.

The four MDGs were significantly upregulated in PACA tissues compared to those in para-cancerous tissues (**Figures 11A–E**). Elevated expressions of these genes were associated with worse outcomes, shorter OS and RFS (**Figures 12A–H** and **Figure S15**). Uni- and multi-variate cox regression analyses indicated that expression levels of GPRC5A and S100A14, as well as TNM stages played important roles in PACA prognosis (**Figures 12I–J**).

## Discussion

There is an urgent need to elucidate the underlying mechanisms of the PACA microenvironment, and to identify novel prognostic biomarkers. Epigenetics, especially DNAm, has shown promise in understanding carcinogenesis, cancer progression and immune surveillance of malignancies (60, 61). However, DNAm is either a driving force of malignancies, or a consequence of genomic deregulation in these malignancies (62). In this study, we found that the methylation loci of the same gene can exert divergent impacts, as evidenced by the heterogeneity and complexity of these epigenetic changes. Therefore, it is important to distinguish between epigenetic changes that promote phenotypes and those “passenger” alterations without any biological effects (63, 64). Moreover, the challenging tasks to interpret their biological effects hindered the utility of pure epigenetic biomarkers.

Identification of MDGs might provide a new horizon for exploring biological effects of epigenetic regulation. Previous studies have employed use of MethylMix as an algorithm for identifying MDGs in diseases. MethylMix requires DNA methylation from normal and disease samples and matched disease gene expression data. Firstly, determination of methylation degree does not rely on arbitrary thresholds. Secondly, identification of a gene as hyper- or hypo-methylated gene is achieved by comparing its differential methylation state between tumorous and normal tissues, and looking for homogeneous subpopulations. Thirdly, matched gene expression data was used to identify transcriptionally predictive DNA methylation events by requiring a negative correlation between methylation and gene expression of a particular gene. Finally, MDGs are selected as methylation possessing a significant predictive effect on their expression, thereby implying that their methylation is predictive of transcription and thus functionally relevant (65). This method has been used to identify MDGs across several cancers, and its reliability has been demonstrated (66, 67). In fact, integrating multi-omics, comprising transcriptomics and epigenomics, is expected to set up a platform for elucidating the underlying mechanisms of PACA (68).

Although immunotherapy has resulted in encouraging response rates in patients with various cancers, its efficacy in PACA patients remain relatively elusive. Accumulating evidence has suggested that the resistance is linked to complex, dual role of the tumor microenvironment, which allows simultaneous PACA promotion and suppression (69). Notably, desmoplastic reaction, a histopathological hallmark for PACA is responsible for the mechanical barrier, where it plays a role in preventing vascularization, thereby limiting exposure to systematic therapies (70, 71). Due to the relative paucity of infiltrated lymphocytes, PACA has been described as a “cold tumor”. However, recent evidence has shown that significant heterogeneity exists in immune cell infiltrates among PACA patients, with a significant positive association between intratumoral CD8+ effector T cell densities and OS (72). In animal models, depletion of Fox3+ regulatory T cells (Tregs) elicited CD8+ T lymphocyte dependent anti-tumor immunity (73). Moreover, inactivated or non-polarized macrophages (M0) have been found to accumulate in various cancers, such as PACA, breast cancer as well as head and neck squamous cell carcinoma (74–76), with a strong association with shortened OS. In addition, not only do tumor associated macrophages (TAMs) and MDSCs directly induce T cell suppression through secreted cytokines (77), they also indirectly induce PDL1 expressions on malignant cell surfaces to inhibit anti-tumor immunities (78). Therefore, success of immunotherapies in PACA patients will rely on elucidating and targeting multiple key steps involved in immune activation, as previously described in the cancer immunity cycle (30). The four-MDG-based signature established in our study will aid in evaluation of immune status and might help stratify patients for immunotherapies.

Compared to other malignancies, such as breast cancer, precision medicine therapies for PACA have not been developed (79). Apart from those well-known somatic mutations in KRAS, TP53, SMAD4 and CDKN2A genes, PACA is highly heterogeneous with several alterations in many other genes, including some epigenetic regulators such as ARID1A, MLL3 and KDM6A (80). Particularly, these genetic alterations converge in intricate core pathways, such as those regulating cell cycle control, epigenetics, as well as WNT/Notch and EGFR signaling pathways, to form PACA hallmarks (81). Despite the low frequencies of most individual genetic mutations, the famous project Know Your Tumor (KYT), in which PACA patients were allocated to matched targeted therapy groups, encouragingly revealed that nearly 40% of all PACA patients harbored at least one genetic alteration that might be therapeutically targeted, while patients who have received matched therapy exhibited significantly longer OS (82). In summary, umbrella design tests aimed at multiplying therapies in different biomarker-matched subgroups have potential benefits for most PACA patients (83). Similarly, our predictive signature has the potential to act as an alternative biomarker for chemotherapy or targeted therapy.

Our prognostic model showed significant correlations with immune cell infiltration and checkpoint expression in PACA, while its risk-scores were positively correlated with TMB. Previous studies have reported that TMB is a useful biomarker for ICB selection, owing to its reflection of overall neoantigen loads (84). Since the cutoffs for categorizing PACA, by TMB stratifications, have not yet been defined, we referred to previous studies (85), and defined TMB-L (low) as ≤5 mut/Mb, TMB-I (intermediate) as ≥6 but <20 mut/Mb, and TMB-H (high) as ≥20 mut/Mb. In the TCGA-PAAD cohort, half of the low-point samples fell into either the TMB-L or TMB-I category, while more than 75% of the high-point samples were TMB-H (**Figures 9A, B****)**, suggesting that our signature might have the potential for identifying new predictive biomarkers for ICB therapy.

It needs to be emphasized that, at the DEG screening stage, we downloaded the merged transcriptomic data of TCGA-PAAD (including 6 normal and 177 tumor) and GTEx pancreas samples (167 normal) from the UCSC-XENA database, in order to increase normal samples and reduce sampling bias. In addition, we selected those intersected MDGs from TCGA-PAAD and ICGC-PACA-AU as candidates, rather than simply integrating these datasets (through “SVA” or other similar packages) into a larger one. We considered the deviances across datasets from irrelevant researches were introduced by various aspects, not just the so-called “batch effects”, in this regard, it should be very cautious to integrate or normalize several individual datasets by some algorithms forcibly.

This study had some limitations. Firstly, both training and validation cohorts had relatively small sample sizes, whereas the platforms and pipelines were not uniform, making the expression values from different cohorts less compatible. Specifically, the cut-off values of the risk-scores across subgroups in each cohort were not uniform. Secondly, although we attempted to evaluate the molecular factors associated with our models, we did not elucidate the underlying mechanisms of action. Thirdly, due to the limited sample sizes, imbalance of clinical features, as well as missing information of the datasets, it was difficult to carry out stratified analyses in our research. Therefore, further studies are needed to unravel these mechanisms.

## Conclusions

In summary, we evaluated MDGs involved in PACA and constructed a four-MDG-based signature for predicting prognosis of PACA patients. Subgroup analyses across all included cohorts revealed that our signature was significantly correlated with immunosuppressive microenvironment in PACA. Specifically, patients with high and low risk-scores might respond differently to chemotherapy, targeted therapy and immunotherapy. Further studies, using more delicate designs and bigger sample sizes, are needed to optimize and validate our predictive model.

## Data Availability Statement

The original contributions presented in the study are included in the article/**Supplementary Material**. Further inquiries can be directed to the corresponding authors.

## Ethics Statement

The studies involving human participants were reviewed and approved by the institutional review board of Zhujiang Hospital. The patients/participants provided their written informed consent to participate in this study. Written informed consent was obtained from the individual(s) for the publication of any potentially identifiable images or data included in this article.

## Author Contributions

YG, GF, FF, and MX designed and surpervised the study, reviewed the manuscript. MX, XJL, and JC analyzed the data and prepared the manuscript. ZY helped revise the manuscript. XJL and YX carried out the histopathological experiments. YZ, YL, XML, and QG helped visualize and validate the results. All authors read and approved the final manuscript.

## Funding

This work was supported by China Postdoctoral Science Foundation (2020M682802), National Natural Science Foundation of China (31972926 and 81800665) and Guangdong Basic and Applied Basic Research Foundation (2020A1515111111).

## Conflict of Interest

The authors declare that the research was conducted in the absence of any commercial or financial relationships that could be construed as a potential conflict of interest.

## Publisher’s Note

All claims expressed in this article are solely those of the authors and do not necessarily represent those of their affiliated organizations, or those of the publisher, the editors and the reviewers. Any product that may be evaluated in this article, or claim that may be made by its manufacturer, is not guaranteed or endorsed by the publisher.

## Supplementary Material

Supplementary Figure 1 Overview of DEGs and differentially expressed MDGs. **(A**, **B)** Expression **(A)** and methylation **(B)** heatmaps of 20 hypomethylated and 10 hypomethylated MDGs in the TCGA-PAAD dataset. **(C**, **D)** Pathway enrichment of DEGs that were up-regulated **(C)** and down-regulated **(D)** in ConcensusPathDB. **(E**, **F)** Pathway enrichment of hypomethylated **(E)** and hypermethylated **(F)** MDGs in ConcensusPathDB.

Supplementary Figure 2 Validation of the four-MDG signature in GSE78229, E-MTAB-6134 and GSE62452 datasets. **(A, D, G)** K-M survival analysis for OS of the high and low risk-score subgroups across the three datasets. **(B, E, H)** Time‐dependent ROC curves of the signature. **(C, F, I)** Risk-score distribution plots across the three datasets. In each plot, from top to bottom: distribution of risk-scores, distribution of survival status, expression patterns of the four genes. **(J**, **K)** K-M analysis for RFS of high and low risk-score subgroups in ICGC-PACA-CA and E-MTAB-6134 datasets.

Supplementary Figure 3 Cox regression analyses for clinicopathological factors in TCGA, ICGC-AU, E-MTAB-6134 datasets.

Supplementary Figure 4 Correlation between PACA clinicopathological features and the four-MDG signature. **(A)** Distributions of risk-scores across four G grades in the TCGA-PAAD, E-MTAB-6134, GSE62452 and GSE78229 databases. **(B)** Distributions of risk-scores across different T stage, N stage, TNM stage and gender subgroups respectively, as well as correlation between patient age and risk-scores in the TCGA-PAAD dataset. **(C)** Distributions of risk-scores across different TNM stage subgroups, and the correlation between patient age and risk-scores in the ICGC-PACA-CA dataset.

Supplementary Figure 5 Features of the four MDGs used to establish our signature. **(A)** K-M curves showing RFS of patients from the TCGA-PAAD cohort based on GPRC5A, SOWAHC, S100A14, ARNTL2 expression. **(B)** Expression levels of the four genes in PACA and normal pancreas samples in the TCGA-PAAD dataset. **(C)** IHC staining of GPRC5A, SOWAHC and S100A14 in PACA tissue from HPA database. **(D)** Genetic alterations in the four MDGs in the TCGA-PAAD dataset from cBioPortal database. Columns and rows indicate tumor samples and MDGs, respectively.

Supplementary Figure 6 PPI network constructed by STRING **(A)** and gene-gene interaction network by GeneMANIA **(B)** of the four signature genes.

Supplementary Figure 7 Immune cells that were significantly correlated with expressions of GPRC5A **(A)**, SOWAHC **(B)**, S100A14 **(C)** and ARNTL2 **(D)** from TIMER 2.0 database.

Supplementary Figure 8 Pearson’s correlation between DNAm levels of the cg sites and mRNA expression of SOWAHC **(A)**, ARNTL2 **(B)** and GPRC5A **(C)**.

Supplementary Figure 9 Functional enrichment of the signature based on GSEA **(A)** and GSVA **(B)** in ICGC-PACA-CA and E-MTAB-6134 datasets.

Supplementary Figure 10 Immune infiltration status of ICGC-AU (seq) dataset. **(A)** Abundances of 22 immune cells by CIBERSORT. **(B)** Upper: correlation between of CD8+ T cell infiltration with the signature; lower: correlation between M0 macrophage with the signature. **(C**, **D)** Immune cell infiltration analysis and immune function enrichment by ssGSEA.

Supplementary Figure 11 Immune infiltration status of ICGC-AU (array) dataset. **(A)** Abundances of 22 immune cells by CIBERSORT. **(B)** Upper: correlation between of CD8+ T cell infiltration with the signature; lower: correlation between M0 macrophage with the signature. **(C**, **D)** Immune cell infiltration analysis and immune function enrichment by ssGSEA.

Supplementary Figure 12 Immune infiltration status of E-MTAB-6134 dataset. **(A)** Abundances of 22 immune cells by CIBERSORT. **(B)** Upper: correlation between of CD8+ T cell infiltration with the signature; lower: correlation between M0 macrophage with the signature. **(C**, **D)** Immune cell infiltration analysis and immune function enrichment by ssGSEA.

Supplementary Figure 13 Profiles of PDCD1 (PD1) and CTLA4 expression patterns in TCGA-PAAD **(A)** and E-MTAB-6134 **(B)** datasets.

Supplementary Figure 14 Differential putative therapeutic responses to dasatinib and bleomycin in TCGA-PAAD **(A)** and ICGC-PACA-AU **(B)** datasets.

Supplementary Figure 15 Associations between MDG proteins and RFS by KM analysis.

Supplementary Table 1 The final 75 differentially expressed MDGs. Sheet 1: The final 75 differentially expressed MDGs. Sheet 2: The gene list of DEGs and MDGs from TCGA-PAAD and ICGC-AU datasets.

Supplementary Table 2 CpG sites of the four signature genes.

Supplementary Table 3 KEGG pathways and biological processes enriched by GSEA.

Supplementary Table 4 KEGG pathways and biological processes enriched by GSVA

Supplementary Table 5 Candidate chemo and targeted drugs identified by the GDSC database in TCGA-PAAD and ICGC-PACA-AU (seq) datasets.

Supplementary Table 6 Candidate compounds identified by CMap analysis in microarray datasets.

Supplementary Table 7 Characteristics of PACA samples collected in our center.
