# Supplementary figures and images for: A DNA-Methylation-Driven Genes Based Prognostic Signature Reveals Immune Microenvironment in Pancreatic Cancer
Source: Front Immunol. 2022 Feb 10;13:803962. doi: 10.3389/fimmu.2022.803962 (PMC8866195; doi:10.3389/fimmu.2022.803962)

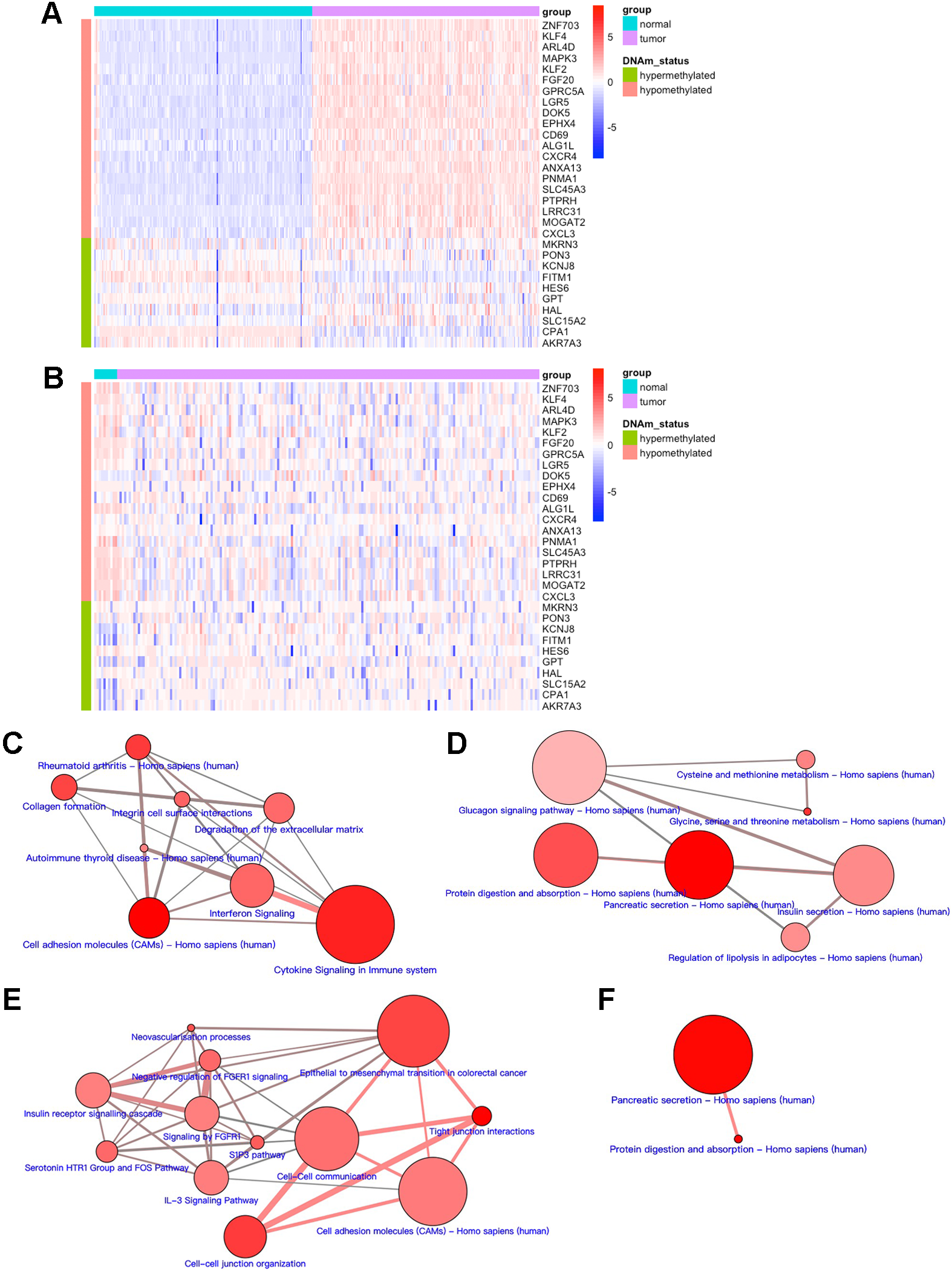

Supplement: Supplementary Figure 1 — Overview of DEGs and differentially expressed MDGs. (A, B) Expression (A) and methylation (B) heatmaps of 20 hypomethylated and 10 hypomethylated MDGs in the TCGA-PAAD dataset. (C, D) Pathway enrichment of DEGs that were up-regulated (C) and down-regulated (D) in ConcensusPathDB. (E, F) Pathway enrichment of hypomethylated (E) and hypermethylated (F) MDGs in ConcensusPathDB. [file Image_1.tif]

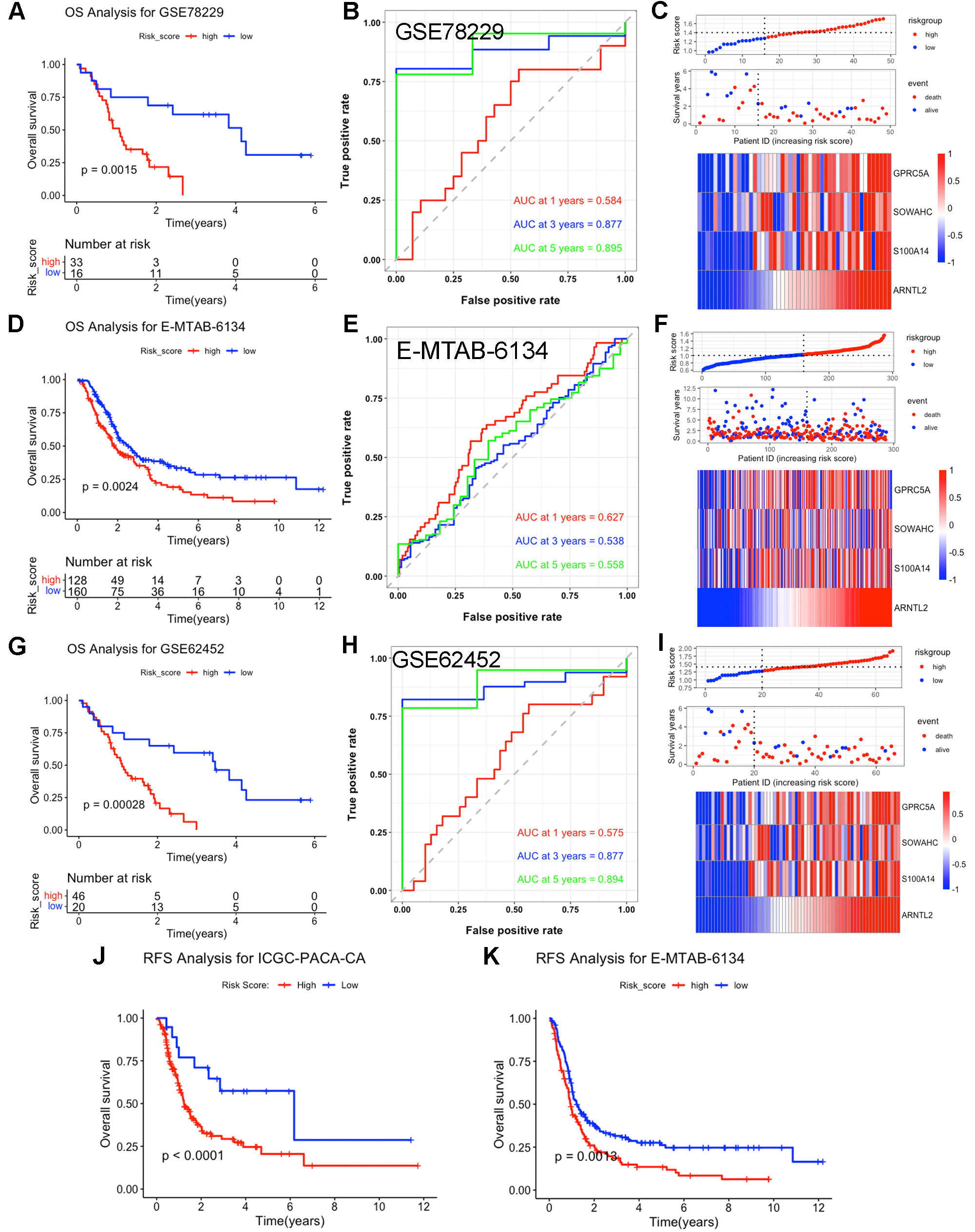

Supplement: Supplementary Figure 2 — Validation of the four-MDG signature in GSE78229, E-MTAB-6134 and GSE62452 datasets. (A, D, G) K-M survival analysis for OS of the high and low risk-score subgroups across the three datasets. (B, E, H) Time‐dependent ROC curves of the signature. (C, F, I) Risk-score distribution plots across the three datasets. In each plot, from top to bottom: distribution of risk-scores, distribution of survival status, expression patterns of the four genes. (J, K) K-M analysis for RFS of high and low risk-score subgroups in ICGC-PACA-CA and E-MTAB-6134 datasets. [file Image_2.tif]

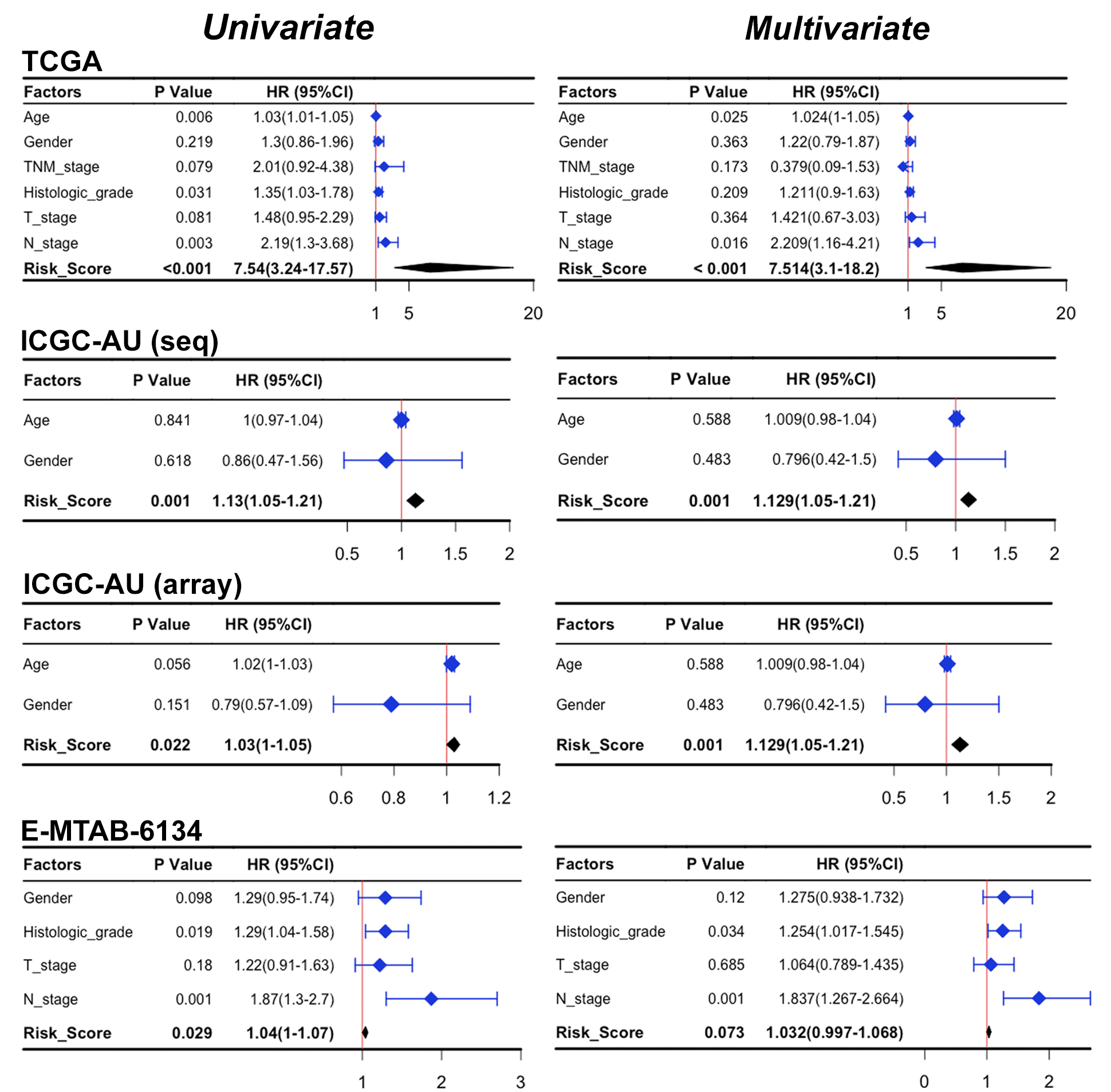

Supplement: Supplementary Figure 3 — Cox regression analyses for clinicopathological factors in TCGA, ICGC-AU, E-MTAB-6134 datasets. [file Image_3.tif]

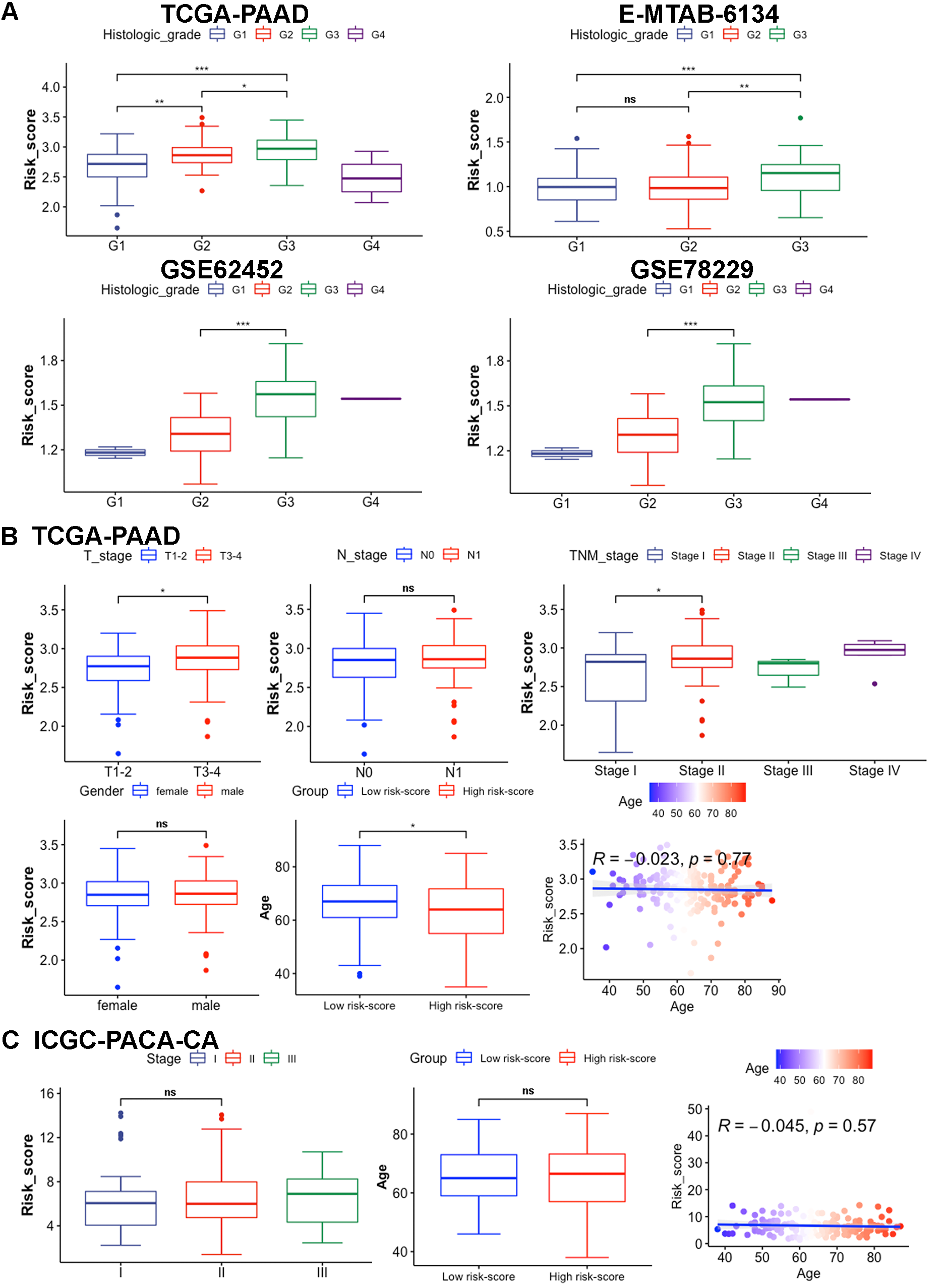

Supplement: Supplementary Figure 4 — Correlation between PACA clinicopathological features and the four-MDG signature. (A) Distributions of risk-scores across four G grades in the TCGA-PAAD, E-MTAB-6134, GSE62452 and GSE78229 databases. (B) Distributions of risk-scores across different T stage, N stage, TNM stage and gender subgroups respectively, as well as correlation between patient age and risk-scores in the TCGA-PAAD dataset. (C) Distributions of risk-scores across different TNM stage subgroups, and the correlation between patient age and risk-scores in the ICGC-PACA-CA dataset. [file Image_4.tif]

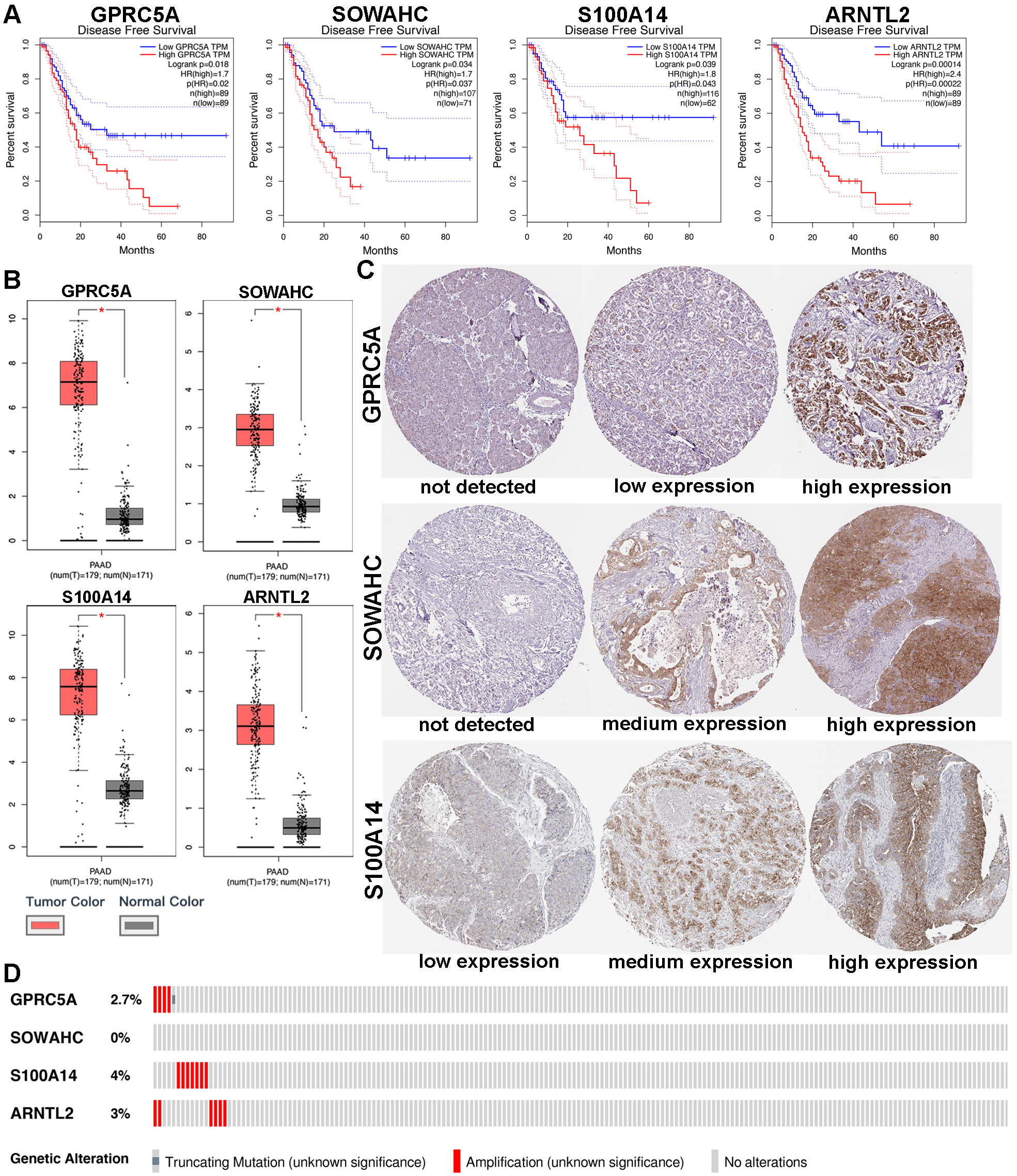

Supplement: Supplementary Figure 5 — Features of the four MDGs used to establish our signature. (A) K-M curves showing RFS of patients from the TCGA-PAAD cohort based on GPRC5A, SOWAHC, S100A14, ARNTL2 expression. (B) Expression levels of the four genes in PACA and normal pancreas samples in the TCGA-PAAD dataset. (C) IHC staining of GPRC5A, SOWAHC and S100A14 in PACA tissue from HPA database. (D) Genetic alterations in the four MDGs in the TCGA-PAAD dataset from cBioPortal database. Columns and rows indicate tumor samples and MDGs, respectively. [file Image_5.tif]

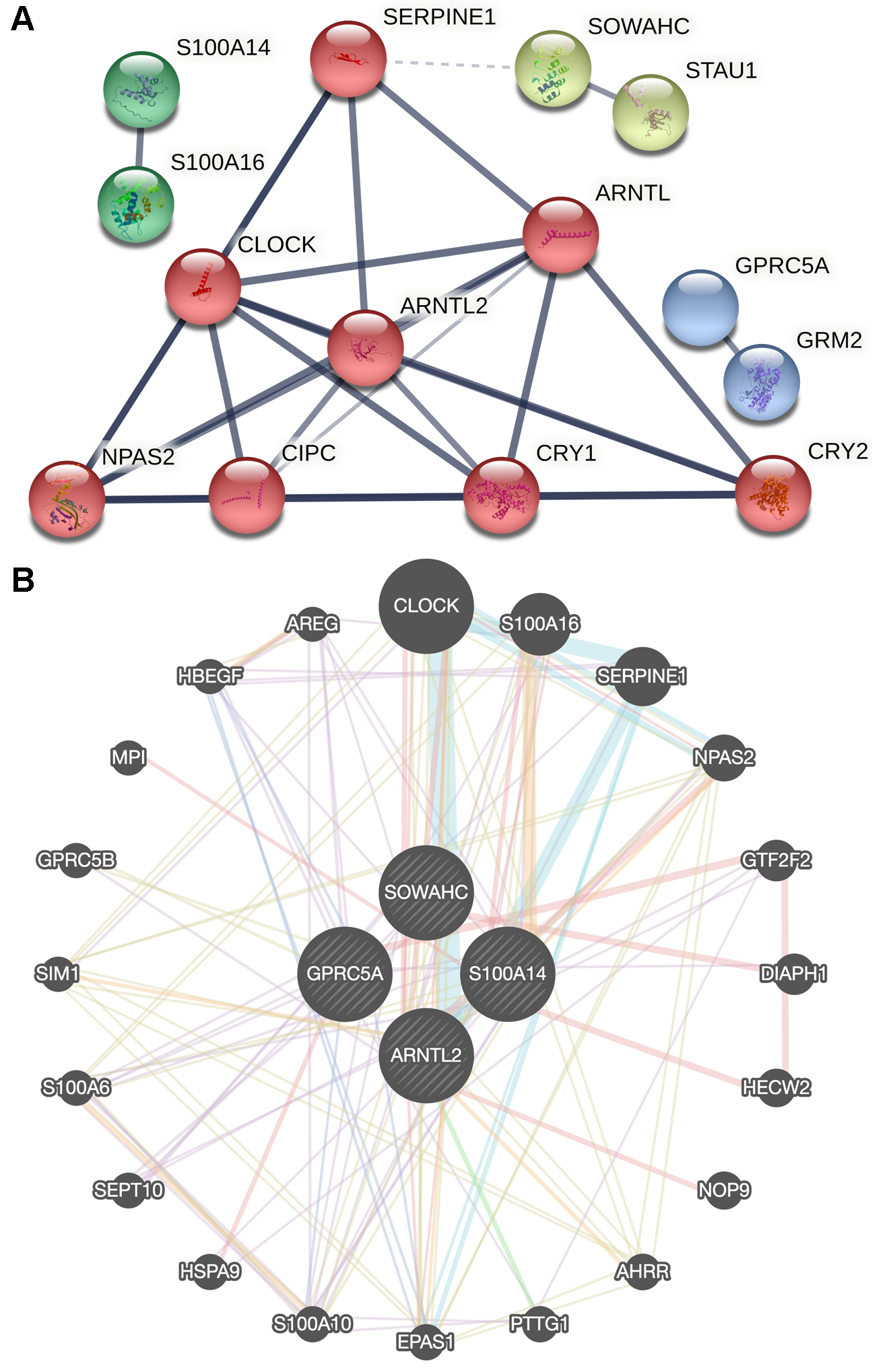

Supplement: Supplementary Figure 6 — PPI network constructed by STRING (A) and gene-gene interaction network by GeneMANIA (B) of the four signature genes. [file Image_6.tif]

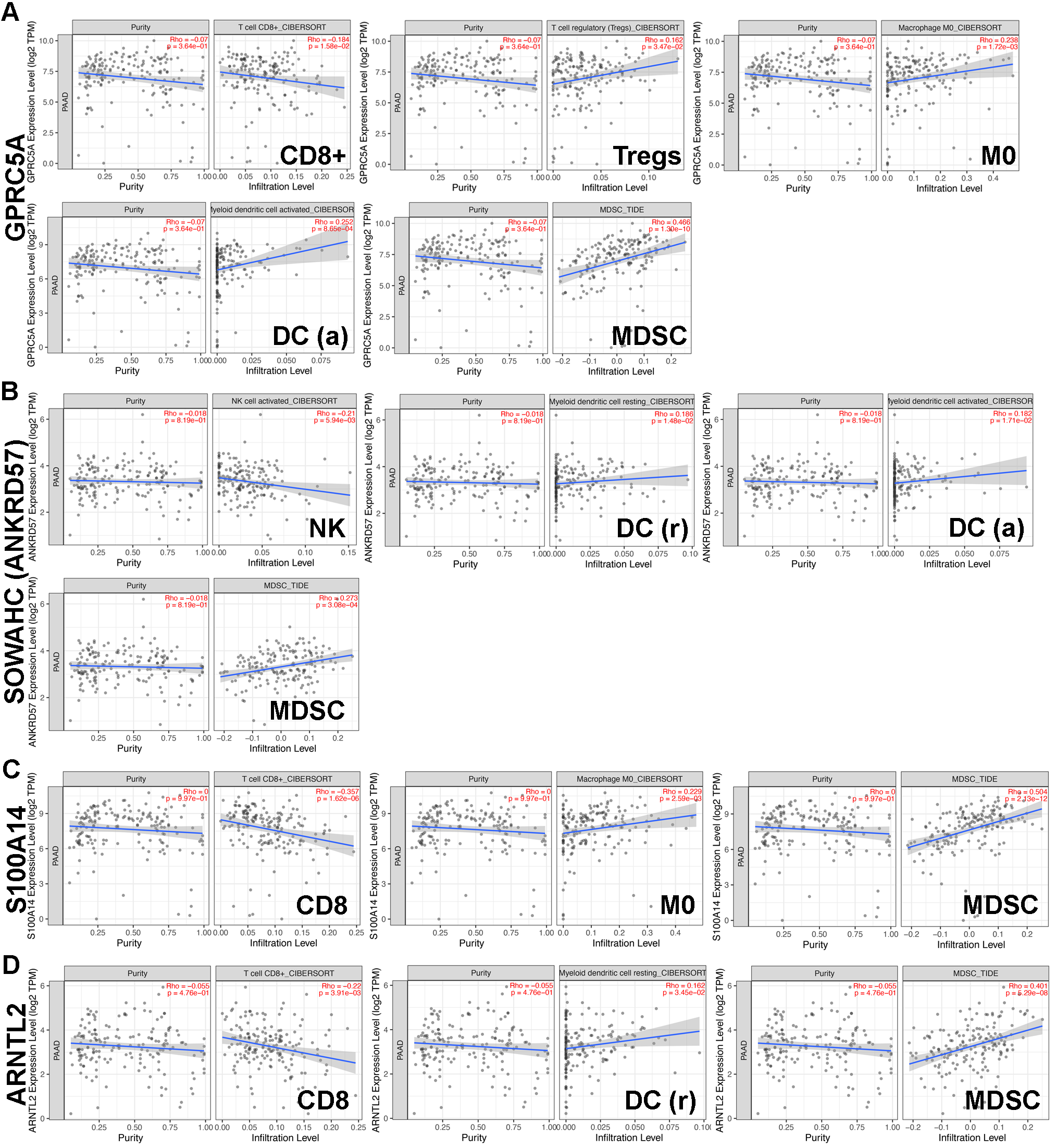

Supplement: Supplementary Figure 7 — Immune cells that were significantly correlated with expressions of GPRC5A (A), SOWAHC (B), S100A14 (C) and ARNTL2 (D) from TIMER 2.0 database. [file Image_7.tif]

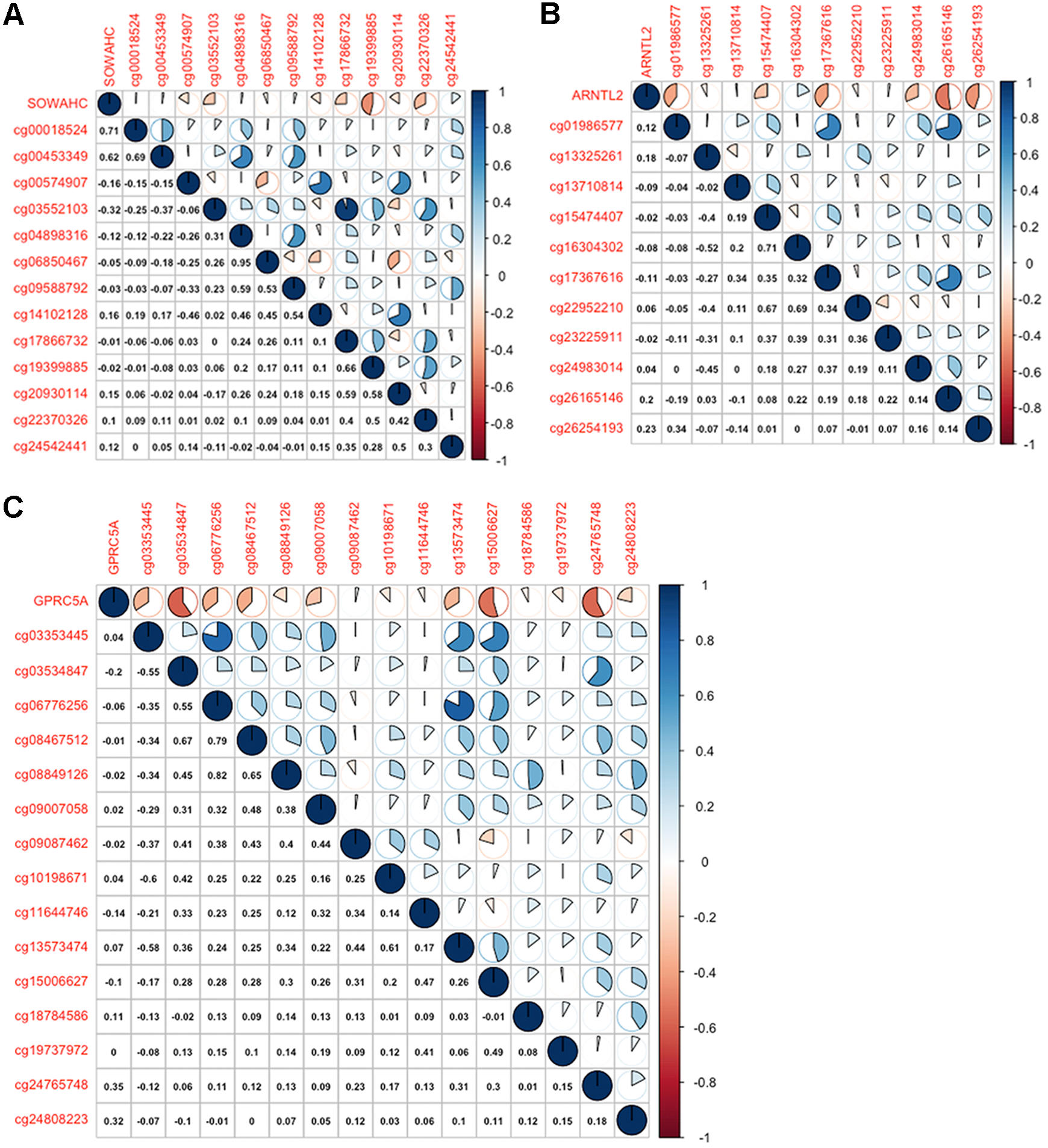

Supplement: Supplementary Figure 8 — Pearson’s correlation between DNAm levels of the cg sites and mRNA expression of SOWAHC (A), ARNTL2 (B) and GPRC5A (C). [file Image_8.tif]

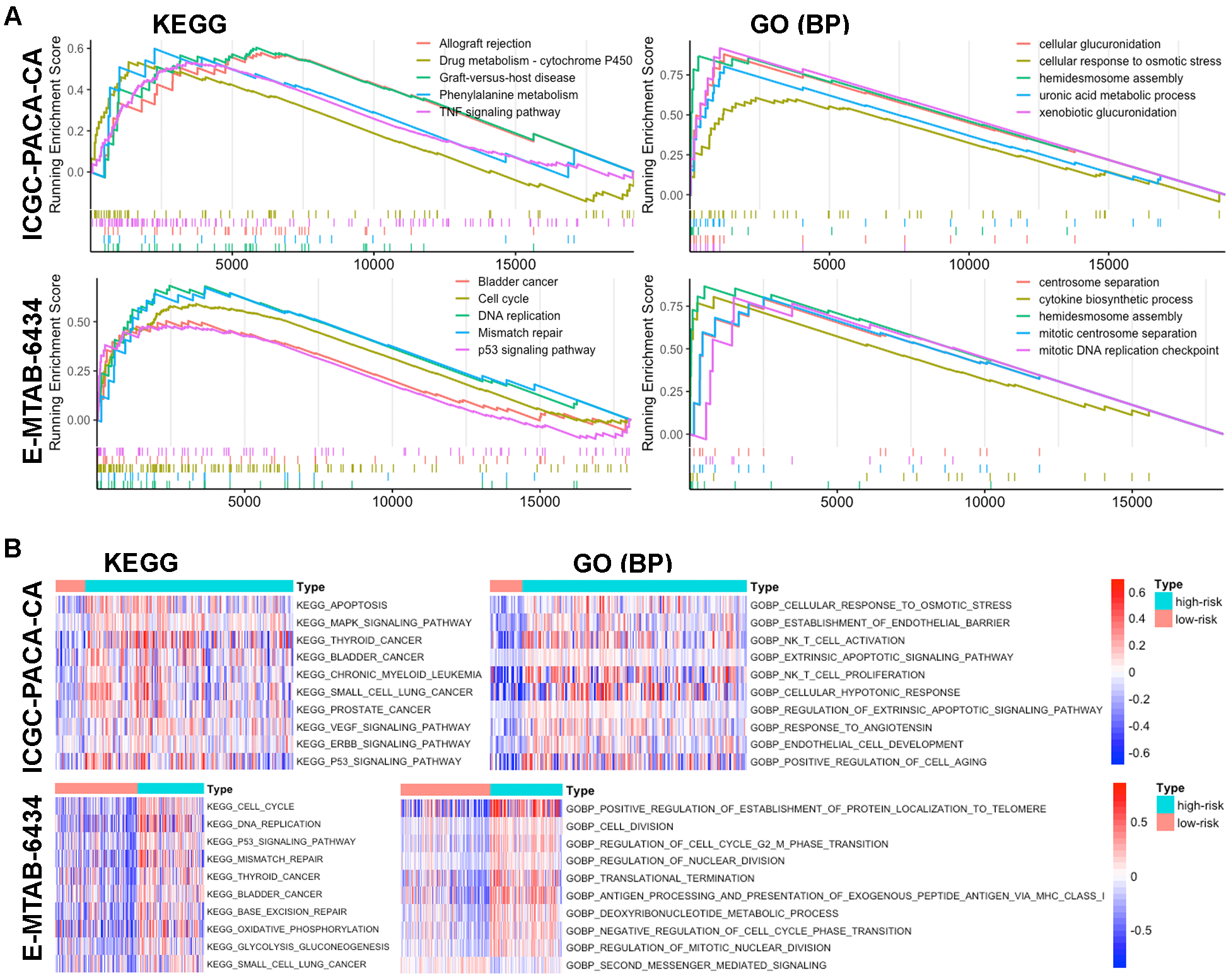

Supplement: Supplementary Figure 9 — Functional enrichment of the signature based on GSEA (A) and GSVA (B) in ICGC-PACA-CA and E-MTAB-6134 datasets. [file Image_9.tif]

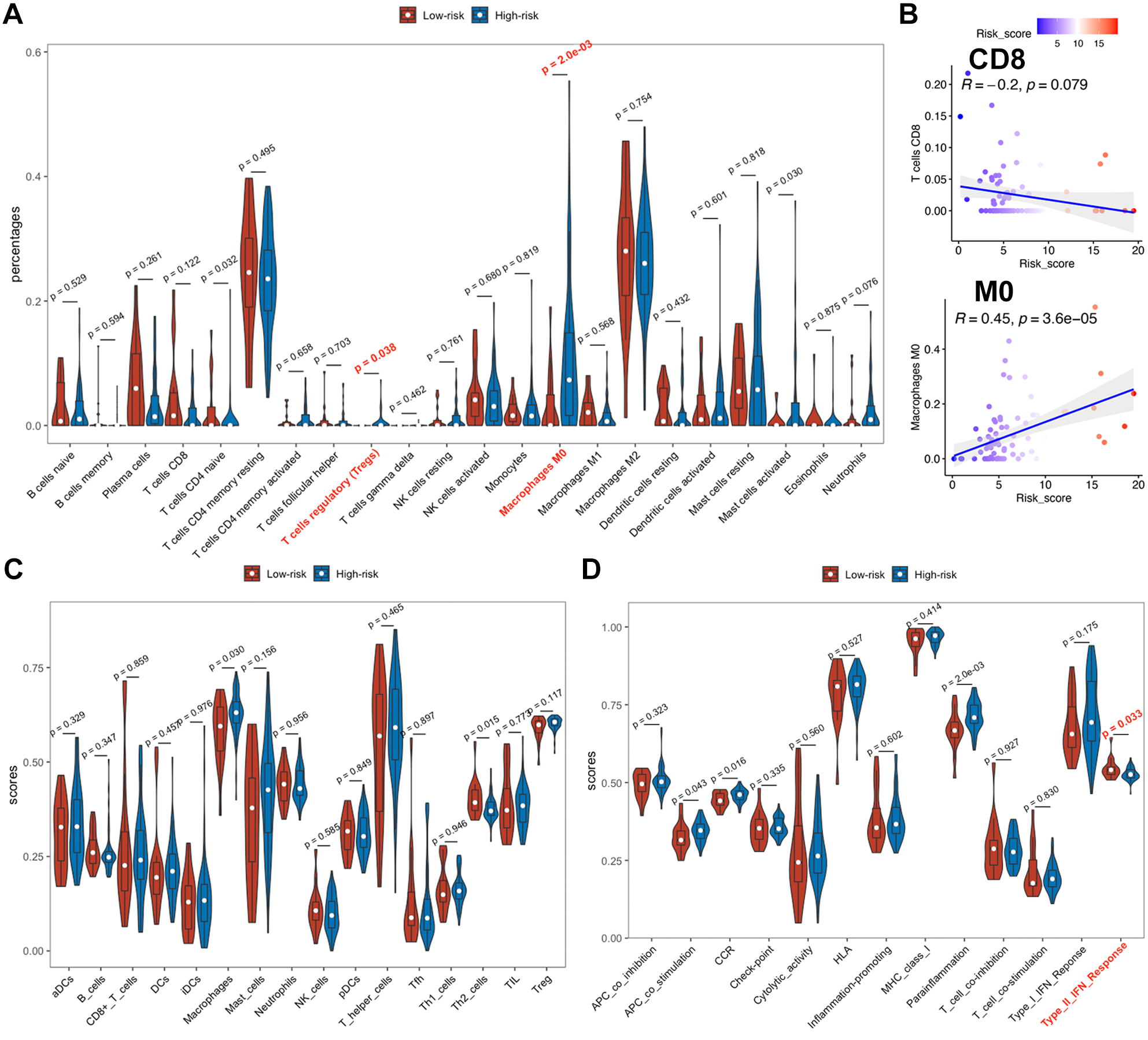

Supplement: Supplementary Figure 10 — Immune infiltration status of ICGC-AU (seq) dataset. (A) Abundances of 22 immune cells by CIBERSORT. (B) Upper: correlation between of CD8+ T cell infiltration with the signature; lower: correlation between M0 macrophage with the signature. (C, D) Immune cell infiltration analysis and immune function enrichment by ssGSEA. [file Image_10.tif]

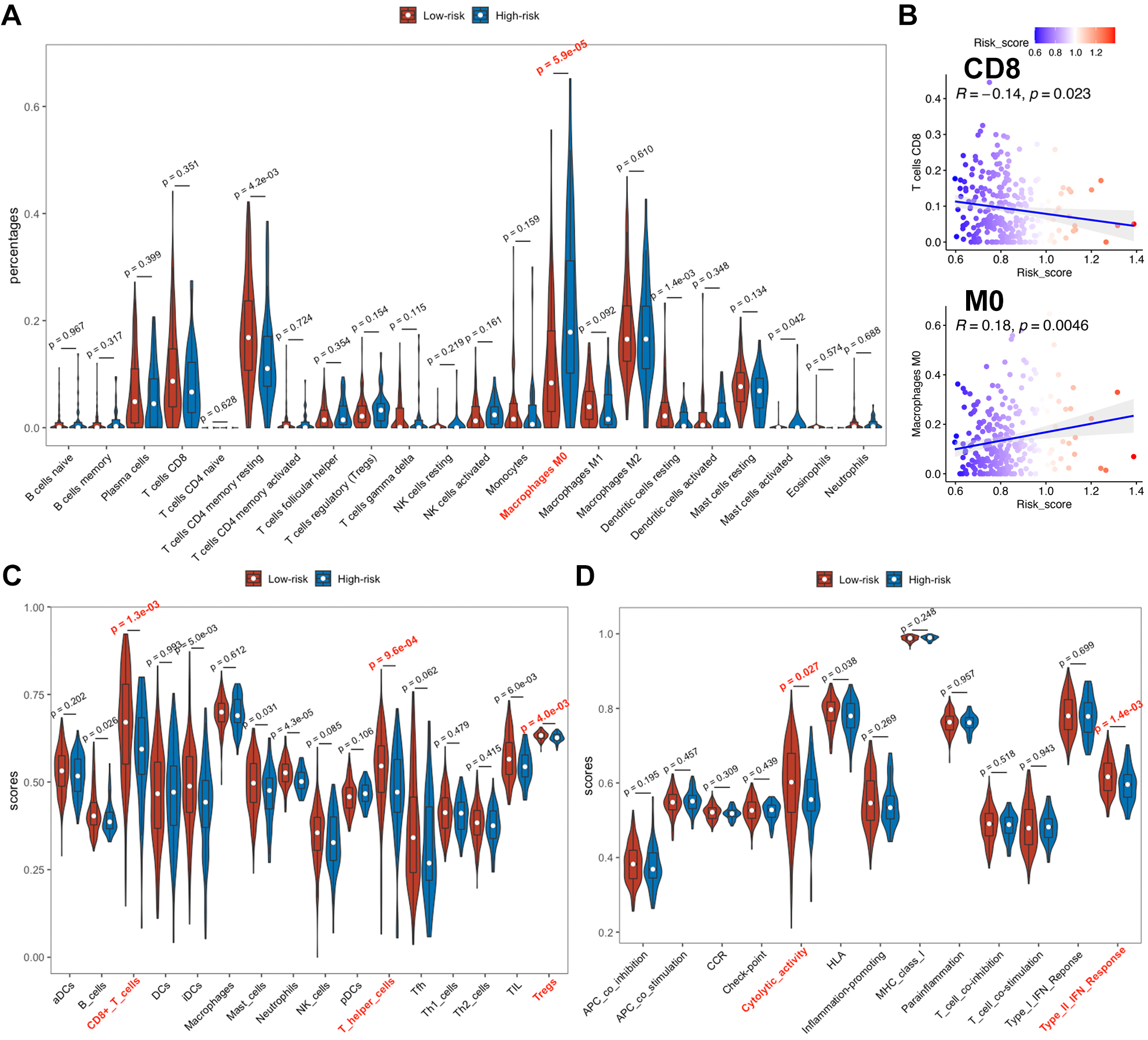

Supplement: Supplementary Figure 11 — Immune infiltration status of ICGC-AU (array) dataset. (A) Abundances of 22 immune cells by CIBERSORT. (B) Upper: correlation between of CD8+ T cell infiltration with the signature; lower: correlation between M0 macrophage with the signature. (C, D) Immune cell infiltration analysis and immune function enrichment by ssGSEA. [file Image_11.tif]

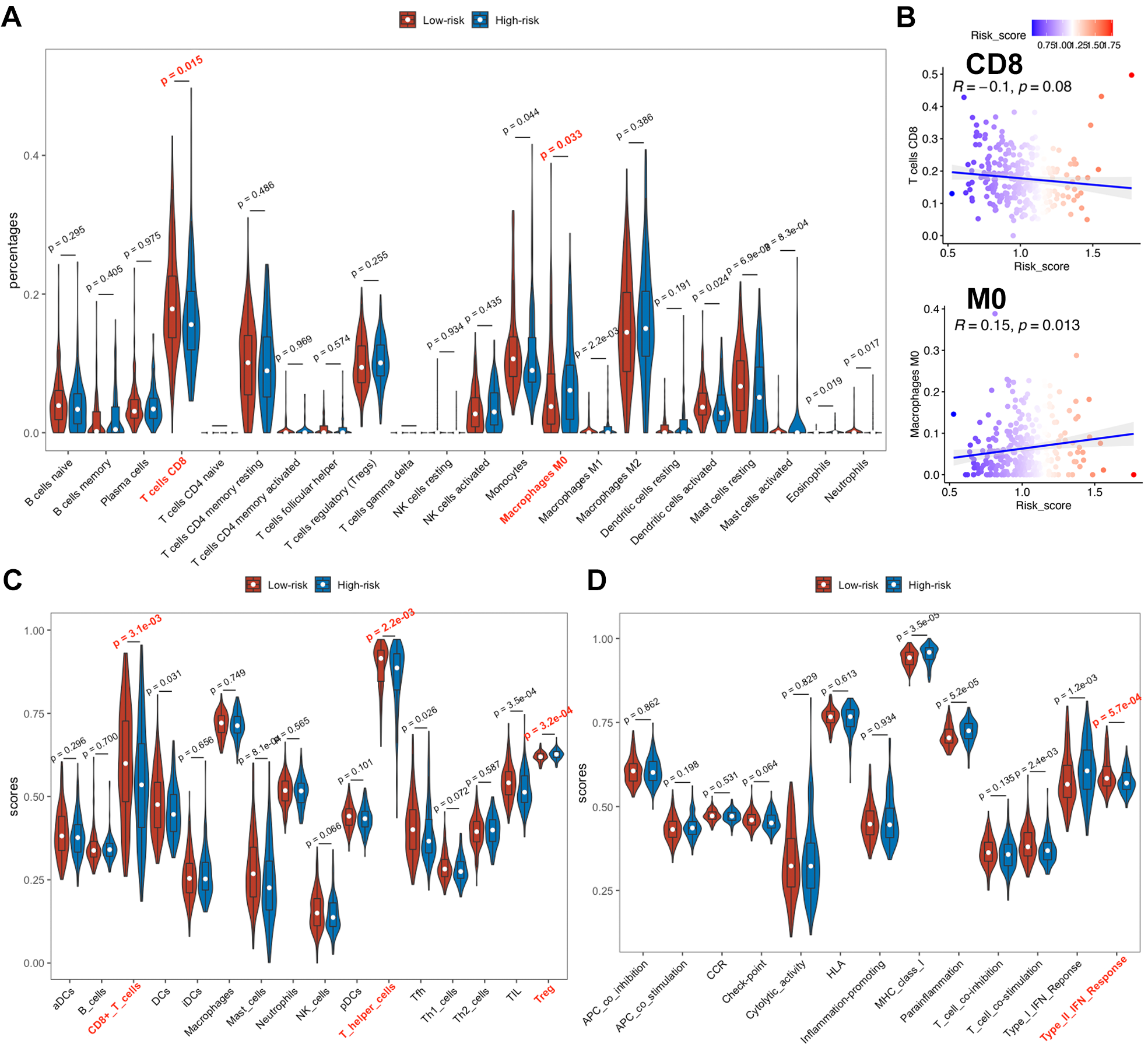

Supplement: Supplementary Figure 12 — Immune infiltration status of E-MTAB-6134 dataset. (A) Abundances of 22 immune cells by CIBERSORT. (B) Upper: correlation between of CD8+ T cell infiltration with the signature; lower: correlation between M0 macrophage with the signature. (C, D) Immune cell infiltration analysis and immune function enrichment by ssGSEA. [file Image_12.tif]

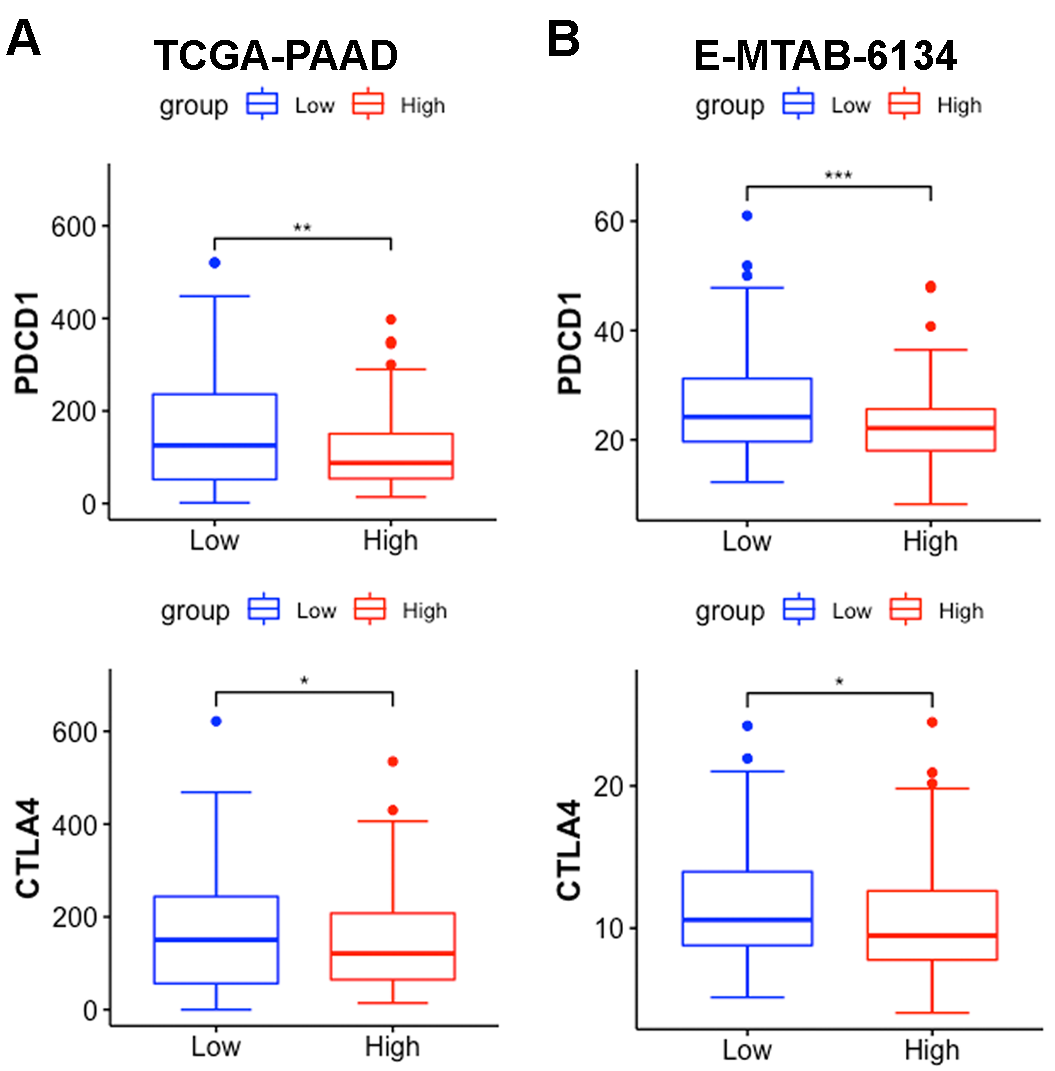

Supplement: Supplementary Figure 13 — Profiles of PDCD1 (PD1) and CTLA4 expression patterns in TCGA-PAAD (A) and E-MTAB-6134 (B) datasets. [file Image_13.tif]

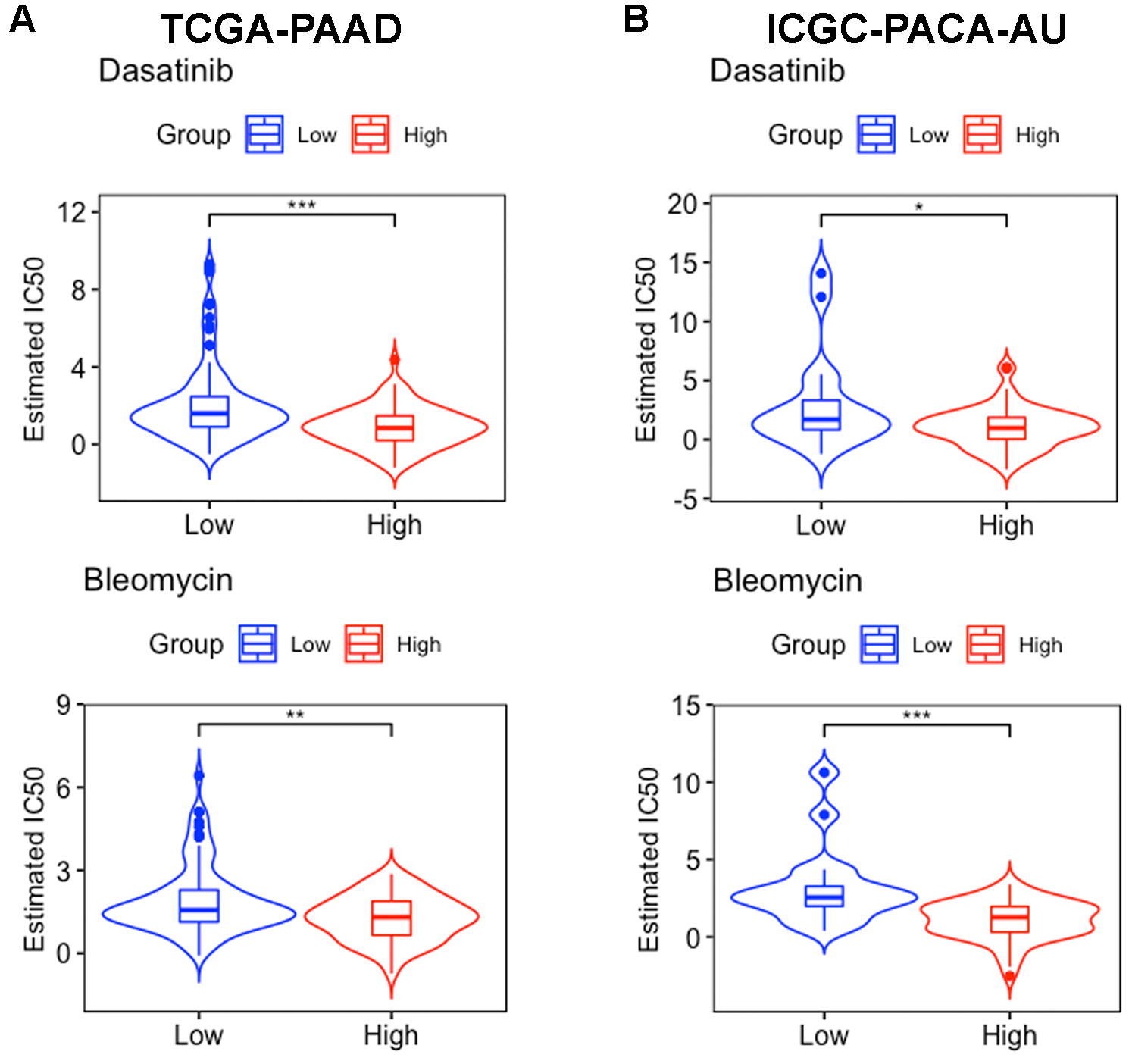

Supplement: Supplementary Figure 14 — Differential putative therapeutic responses to dasatinib and bleomycin in TCGA-PAAD (A) and ICGC-PACA-AU (B) datasets. [file Image_14.tif]

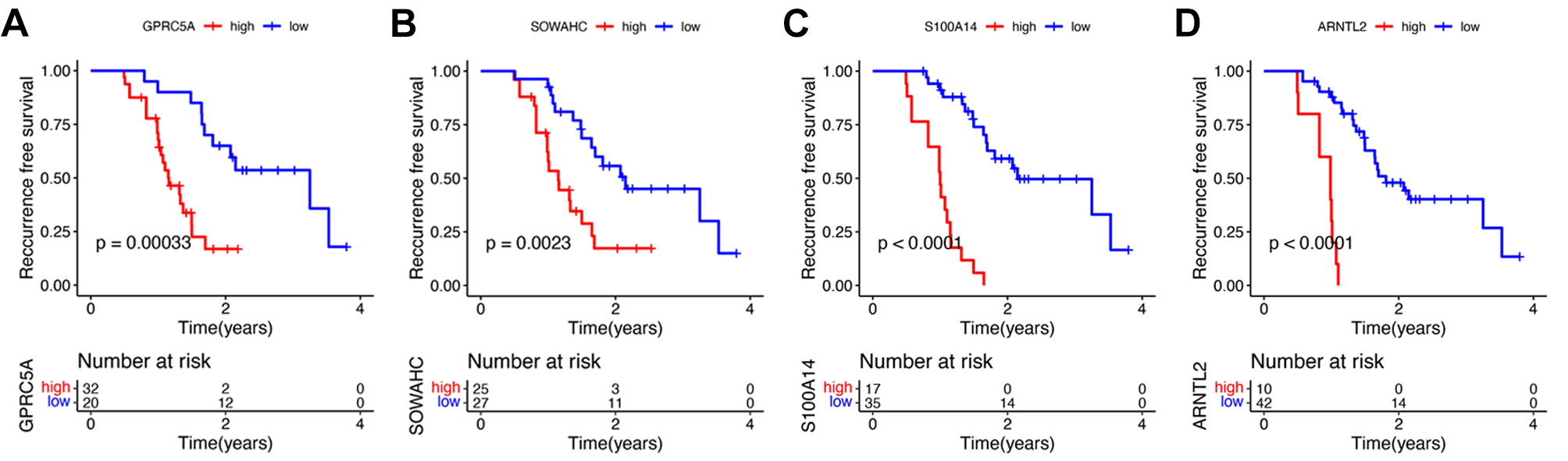

Supplement: Supplementary Figure 15 — Associations between MDG proteins and RFS by KM analysis. [file Image_15.tif]
